# Gm364 coordinates MIB2/DLL3/Notch2 to regulate female fertility through AKT activation

**DOI:** 10.1038/s41418-021-00861-5

**Published:** 2021-10-11

**Authors:** Liang-Jian Chen, Na-Na Zhang, Chun-Xiang Zhou, Zhi-Xia Yang, Yan-Ru Li, Teng Zhang, Cong-Rong Li, Xin Wang, Yang Wang, Zi-Bin Wang, Zheng-Rong Xia, Zhen-Bo Wang, Cui-Lian Zhang, Yi-Chun Guan, Qing-Yuan Sun, Dong Zhang

**Affiliations:** 1 State Key Lab of Reproductive Medicine, Nanjing Medical University, 101 Longmian Ave., Nanjing, 211166 Jiangsu China; 2 Center for Reproductive Medicine, the Third Affiliated Hospital of Zhengzhou University, 7 Rehabilitation Front Street, Zhengzhou, 450000 Henan China; 3 Drum Tower Hospital Affiliated to Medical College of Nanjing University, 321 Zhongshan Road, Nanjing, 210008 Jiangsu China; 4 Reproductive Medical Center, Henan Provincial People’s Hospital & Reproductive Medical Center, People’s Hospital of Zhengzhou University, 7 Weiwu Road, Zhengzhou, 450003 Henan China; 5 State Key Lab of Stem Cell and Reproductive Biology, Institute of Zoology, Chinese Academy of Sciences, 1 Beichen West Road, Beijing, 100101 China; 6 Analysis and Test Center, Nanjing Medical University, 101 Longmian Ave., Nanjing, 211166 Jiangsu China; 7 Fertility Preservation Lab and Guangdong-Hong Kong Metabolism & Reproduction Joint Laboratory, Reproductive Medicine Center, Guangdong Second Provincial General Hospital, 466 Xin-Gang-Zhong Road, Guangzhou, 510317 Guangdong China

**Keywords:** Cell biology, Reproductive disorders, Experimental models of disease

## Abstract

Many integral membrane proteins might act as indispensable coordinators in specific functional microdomains to maintain the normal operation of known receptors, such as Notch. Gm364 is a multi-pass transmembrane protein that has been screened as a potential female fertility factor. However, there have been no reports to date about its function in female fertility. Here, we found that global knockout of *Gm364* decreased the numbers of primordial follicles and growing follicles, impaired oocyte quality as indicated by increased ROS and γ-H2AX, decreased mitochondrial membrane potential, decreased oocyte maturation, and increased aneuploidy. Mechanistically, Gm364 directly binds and anchors MIB2, a ubiquitin ligase, on the membrane. Subsequently, membrane MIB2 ubiquitinates and activates DLL3. Next, the activated DLL3 binds and activates Notch2, which is subsequently cleaved within the cytoplasm to produce NICD2, the intracellular active domain of Notch2. Finally, NICD2 can directly activate AKT within the cytoplasm to regulate oocyte meiosis and quality.

## Introduction

Proper completion of female meiosis is crucial for stable genetic passage, and meiotic abnormalities are implicated in many severe human diseases [1]. Many fertility factors are synthesized and stored during follicle maturation, while the oocytes in the follicles are arrested at the diplotene stage (GV, germinal vesicle stage). Many of these factors are either exclusively or predominantly expressed in ovaries compared to other tissues and are therefore particularly important for oocyte maturation (meiosis) following a surge of luteotropic hormone (LH) [2]. Multiple recent transcriptome- and proteome-wide studies have identified many novel fertility factors; however, their function and regulation remain unknown.

Integral membrane proteins account for ~30% of the total proteome. Little is known, however, about whether or how these proteins function during meiosis, aside from their functions as channels, carriers, or receptors. Some integral membrane proteins have been identified to be essential for meiosis and fertilization of in vitro cultured oocytes. For example, PNMA5 is the most abundant member of the PNMA (paraneoplastic antigen MA) family in oocytes and is enriched on oocyte membranes. Knockdown of PNMA5 results in decreased levels of p-AKT and p-GSK-3β and leads to decreased oocyte quality, unstable meiotic spindle, reduced oocyte maturation, and fertilization [3]. Placenta-specific 1 (PLAC1), which is essential for placentation and implantation, is abundant within oocytes and enriched on the oocyte membrane. PLAC1 knockdown inactivates furin, which cleaves pro-IGF1R into active IGF1R. Thus, PLAC1 knockdown reduces IGF1R level and downstream p-AKT level and subsequently reduces oocyte quality, meiosis, and fertilization [4]. FAM70A is abundantly expressed in oocytes and is enriched on the oocyte membrane. FAM70A directly binds an oocyte-predominant WNT family member, WNT5A, to regulate oocyte quality, meiosis, and fertilization through APC and AKT [5].

Gm364 is a multi-pass transmembrane protein with a conserved EMP70 domain [6] and is, in fact, the only member of the EMP70 family [7]. Although NCBI and BioGPS databases show that Gm364 is predominantly expressed in the testes, neurons, and placenta, no functional data about Gm364 is currently published. It is, however, worth noting that transcriptome-wide screening in *Foxo3a* knockout mouse ovaries identified 13 integral membrane proteins as likely “female fertility factors,” including *Gm364* as well as *Pnma5*, *Plac1*, and *Fam70A* [8]. Foxo3a is a well-studied transcription factor downstream of the mTOR pathway, and it represses the expression of genes required for follicle activation and development. In *Foxo3a* knockout mouse ovaries, almost all follicles undergo synchronous activation, and mice become infertile prematurely (4 months old) [8]. Therefore, genes that were upregulated following *Foxo3a* knockout may be important for follicle activation and development.

In the present study, we show that Gm364 is essential for female fertility and oocyte quality and that it mechanistically operates via regulation of AKT activation through the MIB2/DLL3/Notch2/NICD2 signaling axis.

## Results

### Gm364 is a transmembrane protein essential for female fertility and the quality of follicles and oocytes

Gm364 is a multi-pass transmembrane protein with a conserved EMP70 domain [6, 7]. Although not exclusively expressed in the ovary, Gm364 has been identified as a possible female fertility factor and exhibits an expression pattern typical of maternal factors during embryo development [8]. We were interested to know whether and how Gm364 functions.

**Fig. 1:**
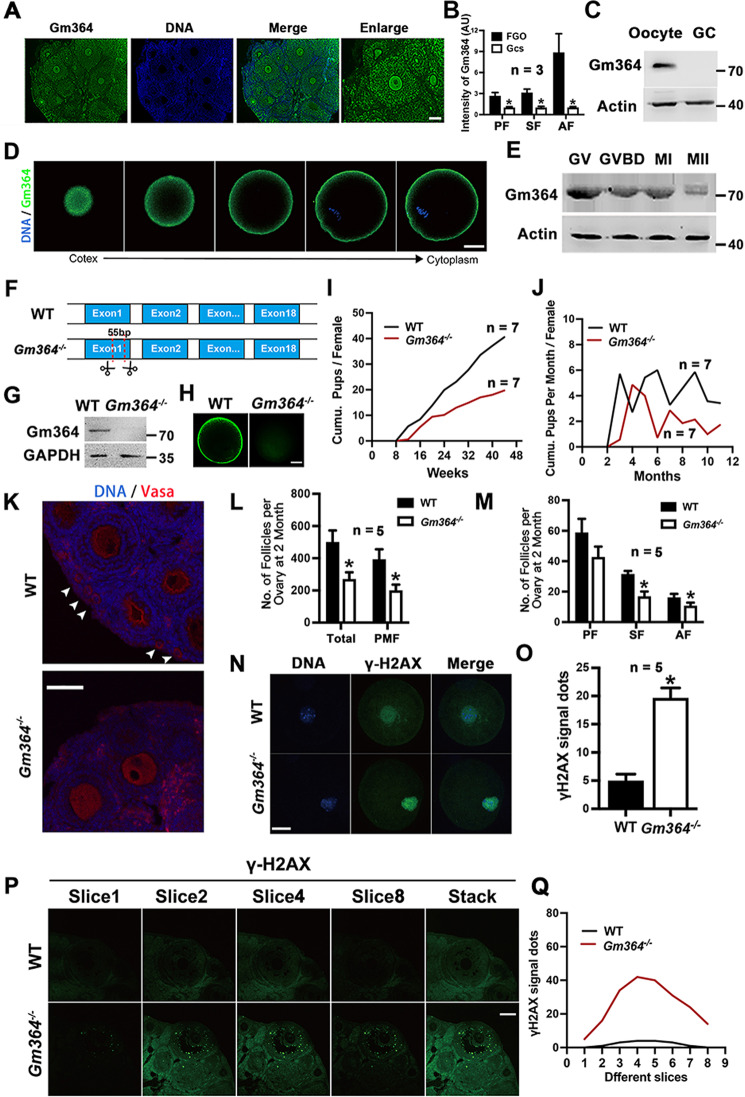
Gm364 is an oocyte-rich transmembrane protein essential for female fertility. **A**, **B** Ovarian immunofluorescence and quantification showed that Gm364 is more abundant in oocytes than in granular cells (GCs), and oocyte Gm364 is more abundant in antral follicles than in primary or secondary follicles. **C** Western blot of oocytes and GCs retrieved from antral follicles showed that Gm364 is more abundant in oocytes than in GCs. **D** Immunofluorescence showed that Gm364 is enriched on the oocyte membrane. Different Z-slices from the cortex to the cytoplasm are shown. DNA in blue, Gm364 in green. **E**. Blots showed that Gm364 protein levels gradually decreased during oocyte meiosis. **F**, **G** Through Cas9 technique, 55 bp was deleted from exon 1 of *Gm364*, which resulted in a frameshift and complete loss of Gm364 protein. **H**. Immunofluorescence showed that Gm364 protein in oocytes almost completely disappeared after *Gm364* knockout. **I**, **J** Fertility assay from 8 to 44 weeks showed that *Gm364* knockout greatly reduced the number of cumulative pups per female (**I**) and the number of cumulative pups per month (**J**). **K**–**M** *Gm364* knockout significantly decreased the numbers of total follicles (TF), primordial follicles (PMF), secondary follicles (SF), and antral follicles (AF). Primary follicles, PF. Primordial follicles are arrow-pointed. DNA in blue, VASA in red. **N**, **O** Immunofluorescence and quantification in oocytes showed that *Gm364* knockout significantly increased γ-H2AX signal dots within oocytes. DNA in blue, γ-H2AX in green. **P**, **Q** Immunofluorescence and quantification in ovaries showed that *Gm364* knockout significantly increased γ-H2AX signal dots within the antral follicles. A series of Z-slices were aligned to show the clear difference (**P**). The number of γ-H2AX signal dots for each slice was counted and aligned into curves (**Q**). β-Actin or GAPDH was used as a loading control. Scale bar for (**A**), (**K**), and (**P**), 100 μm; scale bar for (**D**), (**H**), and (**N**), 20 μm. *Indicates *p* < 0.05.

We performed immunofluorescence and western blot analyses to define the expression and localization patterns of Gm364 during development. We found that Gm364 expression gradually increased as follicles developed from primary follicles to antral follicles (Fig. 1A, B), that it was more highly expressed in oocytes than in granular cells (GCs) in antral follicles (Fig. 1C), and that it was enriched on the oocyte membrane (Fig. 1D). Gm364 levels decreased gradually during in vitro maturation (IVM) (Fig. 1E). These results indicate that Gm364 might function predominantly in oocyte meiosis.

To investigate its function in situ, we used CRISPR/Cas9 targeting to delete 55 bp from exon 1 of *Gm364* (Fig. 1F, supplementary Fig. 1A, B). Western blot and immunofluorescence analyses showed that Gm364 protein was completely absent following *Gm364* knockout (KO) (Figs. 1G and 1H). Fertility assays performed from 8 to 44 weeks showed that *Gm364* knockout greatly reduced the number of cumulative pups per female (Fig. 1I) and the number of cumulative pups per month per female (Fig. 1J). Follicle counting showed that *Gm364* knockout also significantly decreased the numbers of total follicles, primordial follicles, secondary follicles, and antral follicles (Fig. 1K–M). Moreover, immunofluorescence showed that *Gm364* knockout significantly increased γ-H2AX signal dots within fully-grown oocytes (Fig. 1N, O) and antral follicles (Fig. 1P, Q). These findings suggest possible damage to the follicles following *Gm364* knockout.

**Fig. 2:**
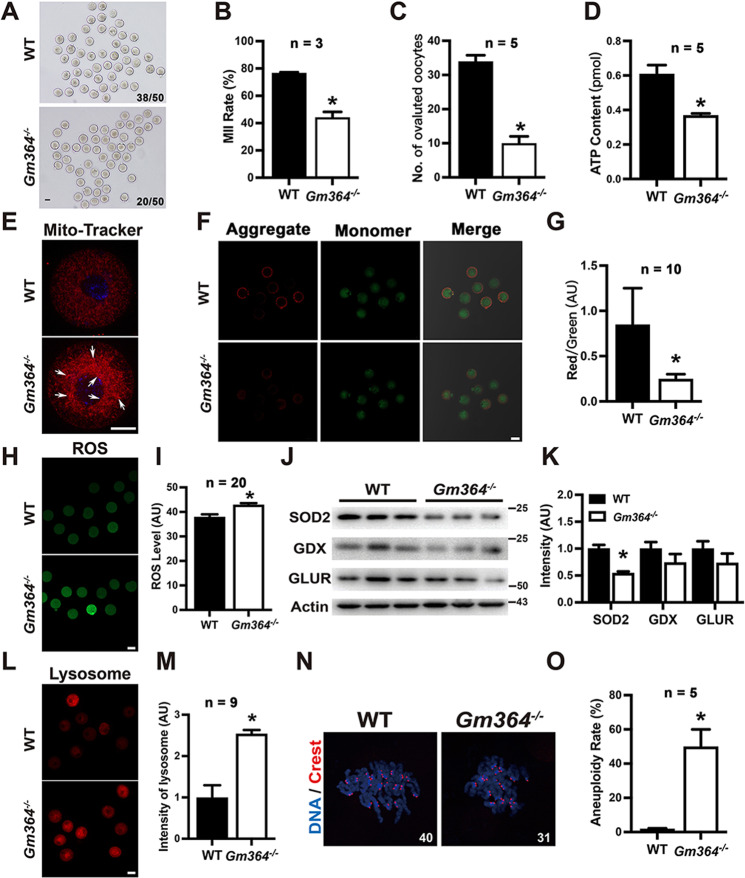
Gm364 is essential for oocyte quality. **A**, **B** *Gm364* knockout significantly reduced oocyte maturation (MII rate). **C** *Gm364* knockout significantly reduced the number of ovulated oocytes. **D** *Gm364* knockout significantly reduced the ATP concentration in oocytes. **E** *Gm364* knockout resulted in mitochondria aggregation (arrow pointed). DNA in blue, mitochondria in red. **F**, **G** *Gm364* knockout significantly reduced mitochondria membrane potential, as indicated by the aggregate (red fluorescence, JC-1 as aggregates at higher membrane potentials) / monomer (green fluorescence, JC-1 as monomers at lower membrane potentials) ratio. **H**, **I** ROS staining and quantification showed that *Gm364* knockout significantly increased ROS levels. **J**, **K** Blot and quantification showed that *Gm364* knockout significantly reduced SOD2 (superoxide dismutase 2) level, but didn’t affect the levels of GDX (glutathione peroxidase) and GLUR (glutathione reductase). **L**, **M** Live fluorescent dye staining and quantification showed that *Gm364* knockout significantly increased lysosome intensity. **N**, **O** Kinetochore staining and quantification of chromosome spread MII oocytes showed that *Gm364* knockout significantly increased aneuploidy. Scale bars for (**A**), (**F**), (**H**), and (**L**), 50 μm; Scale bars for (**E**), 20 μm. *Indicates *p* < 0.05.

As Gm364 is abundant in oocytes and *Gm364* knockout decreased follicle number and increased γ-H2AX signal dots, we next investigated whether oocyte maturation and quality were disrupted. We found that *Gm364* knockout significantly reduced oocyte maturation (MII rate, Fig. 2A, B) and ovulated oocytes (Fig. 2C), indicating that the oocyte quality was affected. We also observed that *Gm364* knockout significantly decreased intracellular ATP levels (Fig. 2D), increased mitochondria aggregation (Fig. 2E, an arrow pointed), decreased mitochondrial membrane potential (Fig. 2F, G), and increased ROS (reactive oxygen species) level (Fig. 2H, I)—altogether suggesting that mitochondria function was severely disrupted. Furthermore, we found that *Gm364* knockout led to a significant decrease in the expression level of SOD2 (Superoxide dismutase 2), although it did not alter the levels of two other antioxidases, glutathione peroxidase (GDX) and glutathione reductase (GLUR) (Fig. 2J, K). We also found that lysosome intensity significantly increased, suggesting that the autophagy level was abnormally high (Fig. 2L, M). Finally, *Gm364* knockout significantly increased the percentage of aneuploidy in MII oocytes (Fig. 2N, O). Taken together, these results indicate that oocyte quality was significantly impaired by *Gm364* knockout.

### Gm364 is essential for Notch2 activation

**Fig. 3:**
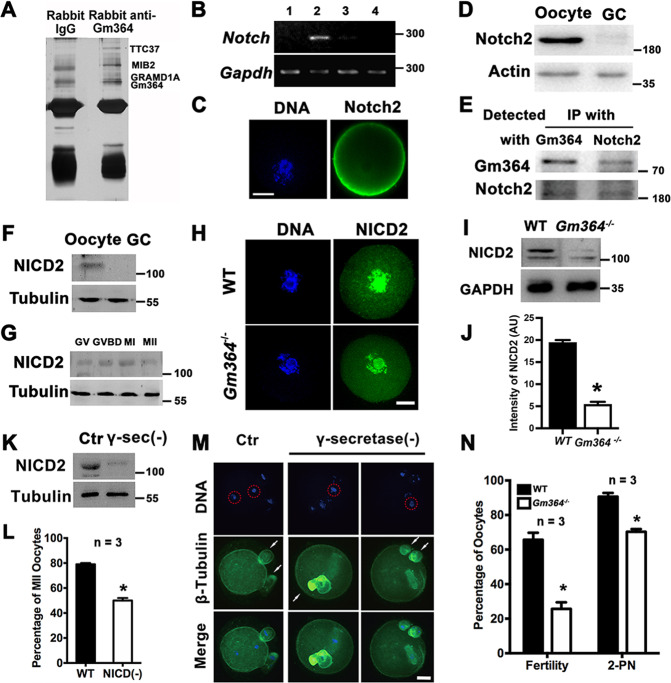
Gm364 is essential for Notch2 activation. **A** Immunoprecipitation with control IgG and Gm364 antibody was performed and followed by SDS-PAGE and silver staining. Then distinct bands were sent for MALDI. TTC37, MIB2, and GRAMD1A were identified as Gm364-interacting proteins. **B** RT-PCR showed that within oocytes, Notch2 is the most abundant among Notch family members 1–4. **C** Immunofluorescence showed that Notch2 was enriched on the oocyte membrane. DNA in blue, Notch2 in green. **D** Western blot showed that Notch2 is more abundant in oocytes than in granular cells. **E** Co-IP and blots showed that Gm364 interacts with Notch2 in oocytes. **F**. Western blot showed that NICD2 was more abundant in oocytes than in granular cells. **G** Blot showed that NICD2 protein levels decreased gradually during oocyte meiosis. **H**–**J** Immunofluorescence and blot showed that *Gm364* knockout significantly decreased the NICD2 protein level. DNA in blue, NICD2 in green. **K** Blot showed that γ-secretase inhibition significantly decreased NICD2 levels. L. NICD2 reduction by γ-secretase inhibition greatly decreased the percentage of MII oocytes. **M**, **N** Immunofluorescence of in vitro fertilized oocytes and quantification showed that inhibiting γ-secretase significantly decreased the percentage of fertilized oocytes and the percentage of 2-PN (two pronucleus). PNs in the control oocyte or chromosomes in the γ-secretase-inhibited (γ-secretase(-)) oocytes were delineated with red dot-line circle; polar bodies (pbs) were labeled with arrows. DNA in blue, tubulin in green. β-actin or α-tubulin was used as a loading control. Scale bar, 20 μm. *Indicates *p* < 0.05.

The data discussed above indicated that Gm364 is important in maintaining the quality of follicles and oocytes. To investigate the functional mechanism of Gm364 activity, we used a Gm364 antibody to perform immunoprecipitation (IP), SDS-PAGE, and silver staining. We then selected bands distinct from the control IgG IP and performed MALDI on them. TTC37, MIB2, and GRAMD1A were identified as Gm364-interacting proteins (Fig. 3A). We first verified that Gm364 co-IPed with each of these three proteins (Supplementary Fig. 2A–D). As MIB1 (mindbomb 1), a closely related member of the mindbomb E3 ubiquitin ligase family, ubiquitinates and activates DLL (Delta-like), the Notch ligands [9], we hypothesized that Gm364 might interact with the Notch pathway. We next showed that *Notch2* mRNA was the most abundant among the Notch family members (Notch1–4) (Fig. 3B) and that Notch2 was also enriched on oocyte membranes (Fig. 3C). Notch2 expression was more abundant in oocytes than in GCs (Fig. 3D), and also co-IPed with Gm364 (Fig. 3E).

Upon binding and activation, Notch proteins are cleaved to generate NICD (Notch intracellular domain) [10]. Similar to Gm364, NICD2 was more abundant in oocytes than in GCs (Fig. 3F) and remained constant during IVM (Fig. 3G). We, therefore, investigated how *Gm364* knockout affects NICD2 and whether NICD2 could also be important for oocyte meiosis. Both immunofluorescence (Fig. 3H) and western blot (Fig. 3I, J) analyses showed that *Gm364* knockout greatly reduced intracellular NICD2 protein levels. Moreover, reduction of NICD2 levels using the γ-secretase inhibitor, RO4929097 (Fig. 3K), greatly reduced the percentage of MII oocytes during maturation (Fig. 3L) and the normal fertilization rate (Fig. 3M, N).

Together, these results indicate that Gm364 could regulate oocyte quality and maturation through Notch2 activation.

### Gm364 regulates MIB2 localization and DLL3 ubiquitination

**Fig. 4:**
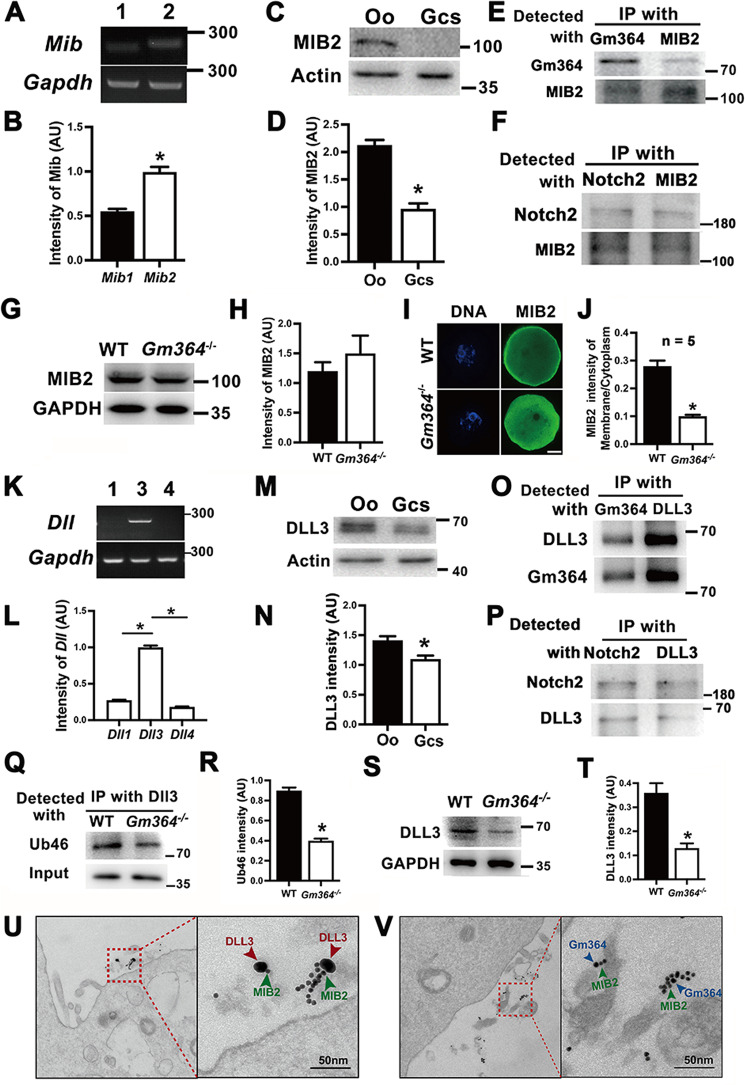
Gm364 regulated the correct states of MIB2 and DLL3. **A**, **B** RT-PCR and quantification showed that *Mib2* mRNA is more abundant than *Mib1* mRNA within oocytes. **C**, **D** Blots and quantification showed that MIB2 is more abundant in oocytes than in granular cells. **E** Co-IP and blot showed that Gm364 interacts with MIB2. **F** Co-IP and blot showed that Notch2 interacts with MIB2. **G**, **H** Blots and quantification showed that *Gm364* knockout did not affect MIB2 protein levels. **I**, **J** Immunofluorescence and quantification showed that MIB2 was enriched on the membrane in WT oocytes, whereas the membrane enrichment completely disappeared and cytoplasmic MIB2 increased in *Gm364* knockout oocytes. **K**, **L** RT-PCR showed that among *Dll* members, *Dll3* was the most abundant in oocytes. **M**, **N** Blots and quantification showed that DLL3 is more abundant in oocytes (oo) than in granular cells (GCs). **O** Co-IP and blot showed that Gm364 interacts with DLL3. **P** Co-IP and blot showed that Notch2 interacts with DLL3. **Q**, **R** DLL3 antibody IP and Ub46 blots showed that *Gm364* knockout significantly decreased DLL3 ubiquitination levels. **S**, **T** Blot and quantification showed that *Gm364* knockout significantly decreased DLL3 protein levels. **U** Immuno-EM showed that MIB2 and DLL3 localized at the oocyte membrane and were close (<20 nm) to each other. MIB2 and DLL3 primary antibodies were bound with 15-nm and 35-nm gold-conjugated secondary antibodies, respectively. **V** Immuno-EM showed that MIB2 and Gm364 localized at the oocyte membrane and were close (<20 nm) to each other. MIB2 and Gm364 primary antibodies were bound with 10-nm and 15-nm gold-conjugated secondary antibodies, respectively. β-Actin or GAPDH was used as a loading control. Scale bar in panel (**I**), 20 μm; scale bar in panels (**U**) and (**V**), 50 nm. *Indicates *p* < 0.05.

We next investigated how Gm364 regulates Notch2 activation. MIB1 is reported to be an E3 ubiquitin ligase that ubiquitinates and activates DLL ligands, which subsequently bind and activate Notch signaling proteins [9]. We found that *Mib2* was more highly expressed than *Mib1* in oocytes (Fig. 4A, B) and that it was also more abundant in oocytes than in GCs (Fig. 4C, D). We, therefore, hypothesized that MIB2 might interact with the Notch2 signaling pathway in oocytes. We found that MIB2 did, indeed, interact with Gm364 (Fig. 4E) and Notch2 (Fig. 4F). Furthermore, we found that although *Gm364* knockout did not alter the levels of MIB2 (Fig. 4G, H) as well as the level of GRAMD1A (Supplementary Fig. 2E, F) and TTC37 (Supplementary Fig. 2G, H), it did completely displace MIB2 from the membrane to the cytoplasm (Fig. 4I, J).

In similar analyses performed on DLL3, we found that *Dll3* mRNA expression levels were the highest among all DLL genes (Fig. 4K, L) and that DLL3 was also more abundant in oocytes than in granular cells (Fig. 4M, N). Next, we observed that DLL3 co-IPed with both Gm364 (Fig. 4O) and Notch2 (Fig. 4P). Moreover, *Gm364* knockout greatly reduced DLL3 ubiquitination levels (Fig. 4Q, R) and protein levels (Fig. 4S, T).

Finally, immuno-EM showed that MIB2 localized close (<20 nm) to DLL3 (Fig. 4U) and Gm364 (Fig. 4V) at the oocyte membrane. Further, Gm364 and all its potential interactors exist only in the soluble fraction of the oocyte membrane (Supplementary Fig. 3). Taken together, these data indicate that Gm364, as an integral membrane protein, can directly bind to and maintain the correct localization of MIB2 to ultimately regulate the ubiquitination of DLL3.

### Gm364 regulates AKT activation through NICD2

**Fig. 5:**
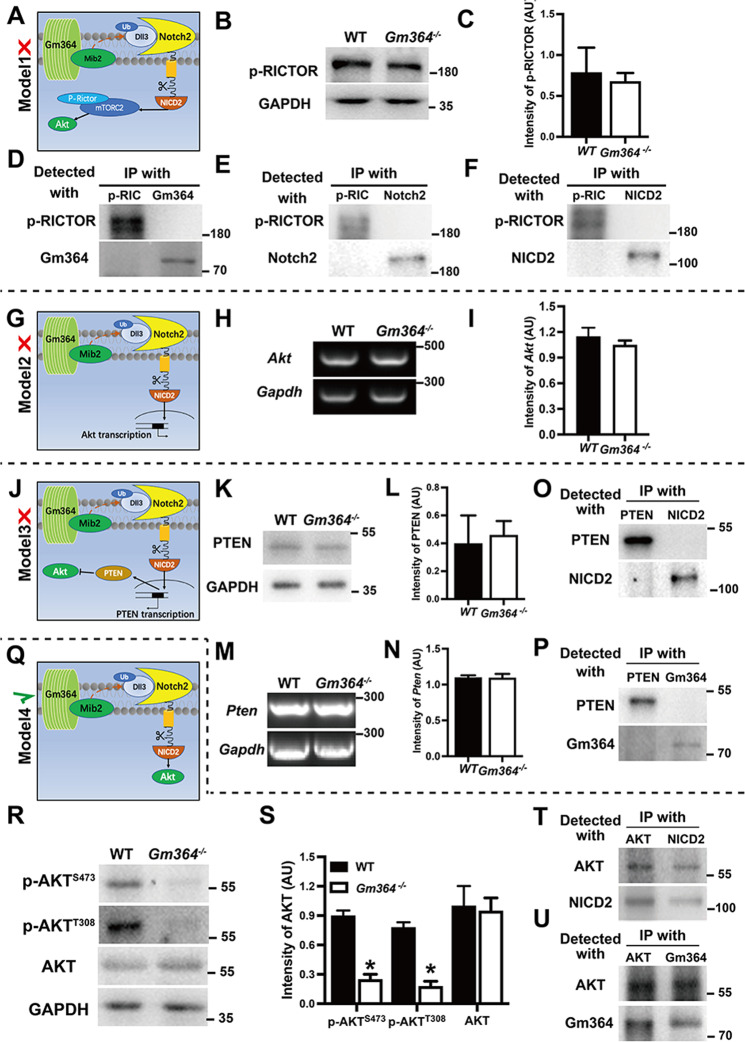
Gm364 regulated AKT activation through NICD2. **A**, **G**, **J**, **Q** To investigate the signal pathway of how Gm364 regulates AKT activation, we created four working models and examined which was the correct pathway. The upstream parts for all four models, i.e., Gm364 binds with MIB2 and DLL3 to promote the cleavage of Notch2 into active NICD2, were the same, while the signals downstream from NICD2 were distinct from each other. **A**–**F** Model 1: if NICD2 first activates mTORC2, then mTORC2 activates AKT, we expected that *Gm364* knockout would significantly decrease p-RICTOR (a component of mTORC2). However, p-RICTOR was unaltered by *Gm364* knockout (**B**) and (**C**), and p-RICTOR did not interact with Gm364 (**D**), Notch2 (**E**), and NICD2 (**F**), suggesting that Model 1 was incorrect. **G**–**I** Model 2: If NICD2 entered the nucleus to promote *Akt* transcription, we expected that *Gm364* knockout would significantly decrease *Akt* mRNA levels. However, *Akt* mRNA was unaltered, suggesting that Model 2 was also incorrect. **J**–**P** Model 3: if NICD2 entered the nucleus to inhibit *Pten* transcription, whereas PTEN negatively regulated AKT, we expected that *Gm364* knockout would significantly increase PTEN protein and mRNA levels. However, neither PTEN protein (**K**) and (**L**) nor mRNA level (**M**) and (**N**) was altered, and neither NICD2 (**O**) nor Gm364 (**P**) interact with PTEN, suggesting that Model 3 was also incorrect. **Q**–**U** Model 4: if NICD2 could directly activate (phosphorylate) AKT, we expected that *Gm364* knockout would significantly decrease p-AKT. Here, *Gm364* knockout significantly reduced AKT phosphorylation at both S473 and T308 (**R**) and (**S**), and both NICD2 (**T**) and Gm364 interact with AKT (**U**), suggesting that Model 4 was correct. GAPDH was used as a loading control.

We next investigated the downstream signals of how Gm364 regulates oocyte meiosis through NICD2. In the preliminary studies before this work, we showed that Gm364 knockout decreased AKT activation. As AKT is a master regulator of oocyte meiosis and quality [11, 12], we formulated four working models based on this and examined which of these models most closely fit with our experimental observations. For Model 1, if NICD2 activates mTORC2, and mTORC2 subsequently activates AKT, we would expect that *Gm364* knockout would significantly decrease p-RICTOR (a component of mTORC2) (Fig. 5A). However, p-RICTOR was unaltered (Fig. 5B, C). In addition, neither Gm364, Notch2, nor NICD2 co-IPed with p-RICTOR (Fig. 5D–F), suggesting that Model 1 is incorrect. For Model 2, if NICD2 translocates to the nucleus to promote AKT transcription, we would expect that *Gm364* knockout would significantly decrease *Akt* mRNA levels (Fig. 5G). However, *Akt* mRNA was unaltered (Fig. 5H, I), suggesting that Model 2 was also incorrect. For Model 3, if NICD2 enters the nucleus to inhibit PTEN transcription, and PTEN negatively regulates *Akt* transcription, we would expect that *Gm364* knockout would significantly increase PTEN protein and mRNA levels (Fig. 5J). However, both PTEN protein (Fig. 5K, L) and mRNA (Fig. 5M, N) levels were unaltered. Furthermore, neither NICD2 (Fig. 5O) nor Gm364 (Fig. 5P) co-IPed with PTEN, suggesting that Model 3 was also incorrect. For Model 4, if NICD2 could directly activate (phosphorylate) AKT within the cytoplasm, we would expect that *Gm364* knockout would significantly decrease p-AKT (Fig. 5Q). Actually, phosphorylation of AKT at both S473 and T308 was significantly decreased by *Gm364* knockout while total AKT was not altered at all (Fig. 5R, S, Supplementary Fig. 4A–C). And further, both NICD2 (Fig. 5T) and Gm364 (Fig. 5U) co-IPed with AKT, and p-Akt^S473^ co-localized with NICD2 (Supplementary Fig. 5A), suggesting that Model 4 could be correct. Finally, we found that Gm364 knockout did not alter the levels of several other important meiotic kinases including p-β-catenin, p-SRC, and p-ERK (Supplementary Fig. 5B, C), which further supports the specificity of NICD2 activation on AKT.

**Fig. 6:**
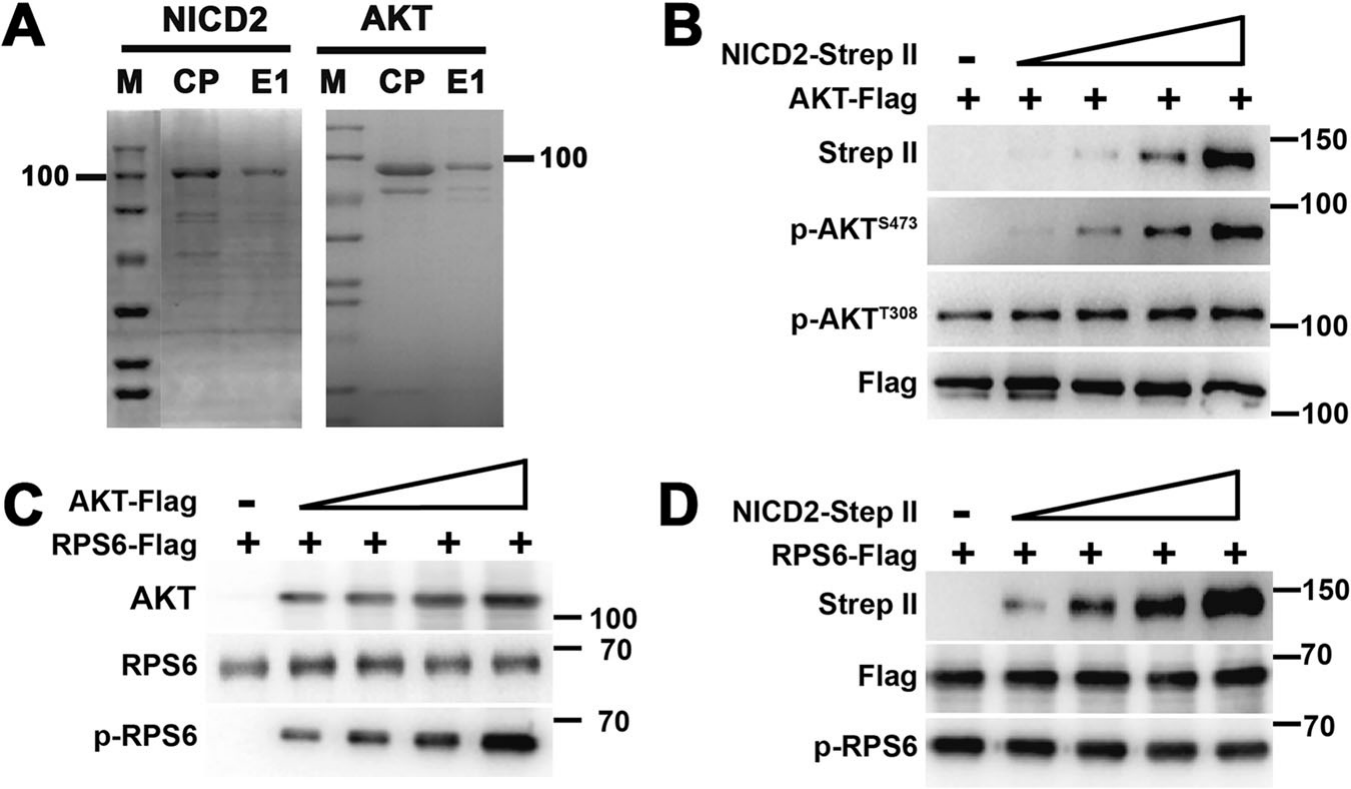
NICD2 specifically regulated AKT activation in vitro. **A** NICD2-strep II and AKT-flag were expressed and purified through the Bac-to-bac system. SDS-PAGE and commassie staining showed that the final purified proteins had good purity. **B** In vitro phosphorylation and blot showed that as NICD2 increased, both p-AKT^S473^ and p-AKT^T308^ levels increased, while p-AKT ^S473^ increased more dramatically. **C**, **D** In vitro phosphorylation and blot showed that as AKT increased, p-RPS6 level significantly increased (**C**); however, NICD2 increment didn’t affect p-RPS6 at all (**D**).

We next tried to verify that NICD2 directly binds and activates AKT using in vitro analysis. We used the Bac-to-bac system to clone and express NICD2 and AKT and subsequently purified the two proteins using Ni-NTA Superflow resin (Fig. 6A). An in vitro phosphorylation assay showed that the phosphorylation of purified AKT (RFP-FLAG-tagged) alone was absent at S473 and low at T308, while as NICD2 (EGFP-Strep II) increased, AKT phosphorylation at S473 rapidly increased in a dose-dependent manner (Fig. 6B). AKT phosphorylation at T308 also increased, but to a lesser extent (Fig. 6B). We also showed that although AKT could phosphorylate RPS6 in a dose-dependent manner (Fig. 6C), NICD2 could not (Fig. 6D), which further supported the specificity of NICD2 on AKT. These results indicate that Gm364 regulates AKT activation through NICD2.

It is known that AKT activity is essential for normal meiosis, including in vitro cultured oocytes [11, 12]. We demonstrated that Gm364 knockdown through siRNA (Supplementary Fig. 6A–D) also decreased oocyte maturation (Supplementary Fig. 6E–G), NICD2, and p-AKT level (Supplementary Fig. 6H, I), while AKT inhibition by the specific inhibitor, Perifosine (Supplementary Fig. 6J, K), also significantly decreased oocyte maturation (Supplementary Fig. 6L, M). These data indicate that Gm364 might initiate an autocrine pathway to regulate meiosis through NICD2-AKT activation.

### Gm364 is essential for normal embryo development

**Fig. 7:**
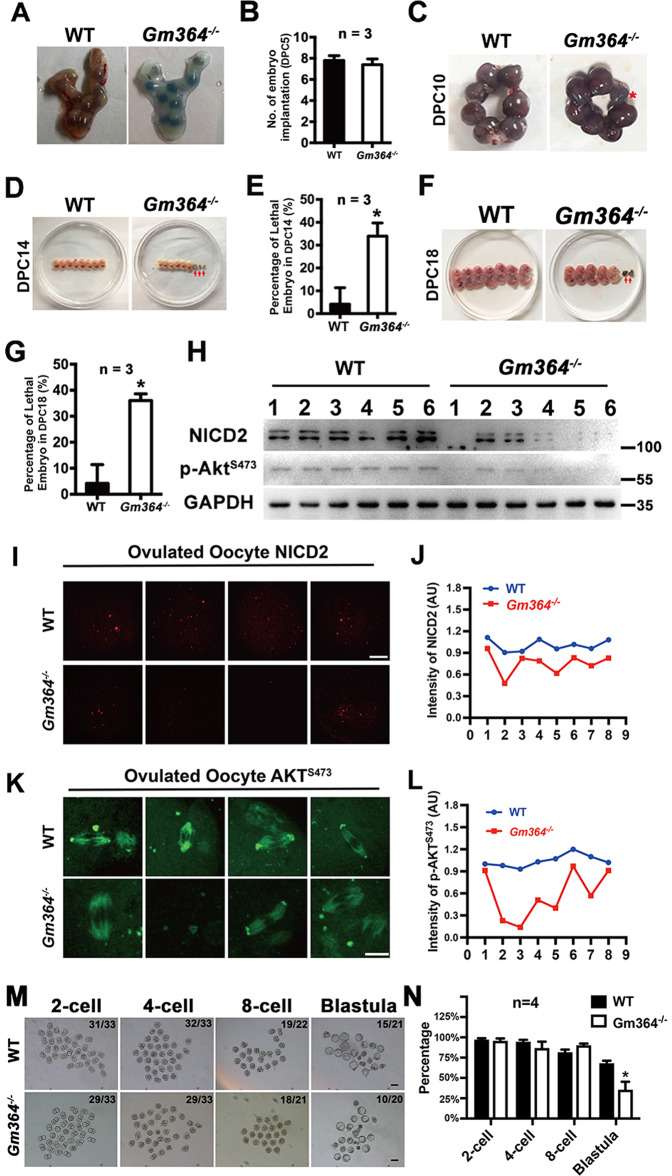
Gm364 is essential for normal embryo development. **A**, **B** Trypan blue staining and quantification showed that *Gm364* knockout did not change the number of implanted embryos at DPC (days post coitum) 5. **C** At DPC 10, some implanted embryos were abnormally smaller (red asterisk). **D**–**G**. Morphological evaluation and quantification showed that, at DPC 14 (**D**) and (**E**) or DPC 18 (**F**) and (**G**), *Gm364* knockout mice had a significantly larger percentage of tiny dead embryos (red arrow) than the WT mice. **H** Blots showed that, at DPC 21, a large percentage (4 of 6, red arrows) of *Gm364-*KO mouse embryos had significantly lower NICD2 and p-AKT^S473^ levels; In contrast, all WT embryos had higher and similar NICD2 and p-AKT^S473^ levels. **J**–**L** Immunofluorescence and quantification showed that NICD2 levels (**I**) and (**J**) or p-AKT^S473^ (**K**) and (**L**) are all higher and close to each other in ovulated WT oocytes (blue curves); while lower and very distinct from each other in ovulated *Gm364-*KO oocytes (red curves). **M**, **N** In vitro culture of in vivo fertilized oocytes from 2-cell to blastula showed that the blastula rate of *Gm364-*KO fertilized oocytes (35.5%) was significantly less than that of WT fertilized oocytes (68.1%). Scale bars for panel (**I**), 20 μm; scale bars for panel (**K**), 10 μm; Scale bars for panel (**M**), 100 μm. *Indicates *p* < 0.05.

As *Gm364* knockout decreased oocyte maturation and quality, we expected that it would also affect embryo development. We, therefore, closely analyzed embryo development at different time points. At 5 days post coitum (DPC), there was no difference in implanted embryos between the control and *Gm364*-KO mice (Fig. 7A, B). However, at 10 DPC some implanted embryos within the uterus were significantly smaller (Fig. 7C, red star); and at 14 DPC (Fig. 7D, E) or 18 DPC (Fig. 7F, G) significantly more embryos dissected from the uterus were obviously abnormal developmentally (absorbed). Next, we examined the NICD2 and p-AKT levels within multiple WT and *Gm364*-KO ovaries in parallel. We found that, although NICD2 and p-AKT levels decreased within all *Gm364*-KO ovaries, the extent of this decrease differed (Fig. 7H). This could explain our observation that in the *Gm364*-KO group, some embryos were normal and the normally delivered post-natal mice were physiologically normal, while other embryos were obviously abnormal.

To verify that the normally delivered *Gm364*-KO mice were, indeed, physiologically normal, we first determined that there were no differences in either the body weight or weight of major organs between WT and *Gm364-*KO female mice (Supplementary Fig. 7A, B). Secondly, we examined multiple key blood biochemical indexes and found that there was no significant difference for any of the indexes between the WT and *Gm364*-KO groups (Supplementary Fig. 7C–N). We also examined several immunity and inflammation markers including CRP (C-reactive protein), CD4 (cluster of differentiation 4), IL (interleukin) 4, and IL6 in the liver (Supplementary Fig. 8A, B) and kidney (Supplementary Fig. 8C, D), and found no significant differences between WT and *Gm364*-KO animals. Finally, we examined major apoptosis and proliferation markers including Bax/Bcl2 (Supplementary Fig. 9A–D) and Ki67 (Supplementary Fig. 9E–H) in the kidney and liver and did not find any significant differences.

These findings indicate that Gm364 is essential for embryo development, but only some of the *Gm364*-KO embryos developed abnormally due to a severe decrease in NICD2 and p-AKT. These results also highlight that Gm364 primarily affects the ovaries and oocytes. To further verify that the oocytes in *Gm364*-KO ovaries have larger discrepancies than the oocytes from WT mice, we collected ovulated oocytes from WT or *Gm364*-KO mice picked at random and stained for expression of NICD2 and AKT. We observed that the intensity of either NICD2 (Fig. 7I, J) or p-AKT^S473^ (Fig. 7K, L) staining was significantly more variable in *Gm364*-KO oocytes than in WT oocytes. Next, we analyzed the in-vitro development rate of in-vivo fertilized oocytes from the 2-cell to blastula stage. We found that 68.1% of fertilized WT oocytes could develop to blastula, whereas only 35.5% of fertilized *Gm364*-KO oocytes could develop to blastula. This, together with the abnormal development in *Gm364*-KO embryo from 10 DPC to 18 DPC, supported that *Gm364* knockout severely impedes the whole embryonic development process.

### *Gm364* Knockout significantly alters the transcription and DNA methylation profiles of oocytes

**Fig. 8:**
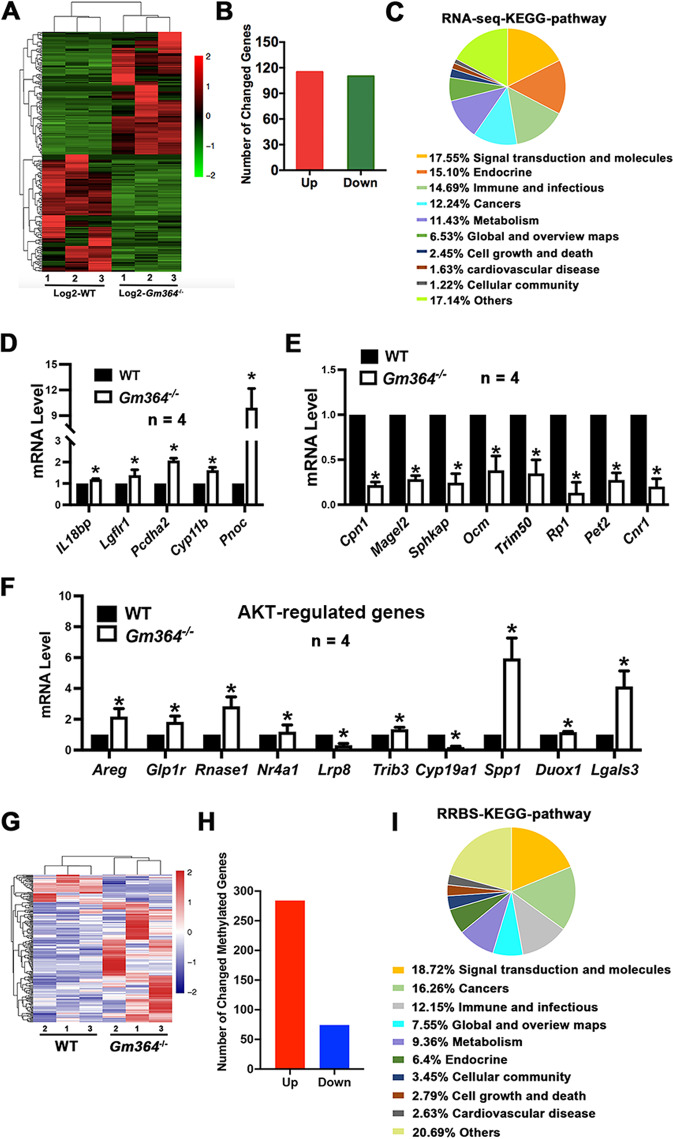
*Gm364* knockout significantly changed the DNA transcriptional and methylational profiles. **A** RNA sequencing of WT and *Gm364-*KO PND 21 ovaries showed that many genes, including both function-annotated and function-unannotated genes, were differentially expressed. **B** Focusing on the function-annotated genes showed that there were 225 differentially expressed genes (DEGs) at the folding threshold of |log2[*Gm364*-KO/WT]|>2; among them, 51% (115 of 225) were upregulated, and 49% (110 of 225) were downregulated. **C** KEGG analysis showed that the top three groups of genes were involved in signal transduction, endocrine, and immunity and infections. **D**, **E** Q-PCR verification of the up-regulated (**D**) and down-regulated (**E**) DEGs in ovarian RNA sequencing. **F** Q-PCR verification of AKT-regulating genes. **G** RRBS on PND 21 ovaries showed that the promoter regions of many genes, including both function-annotated and function-unannotated genes, were differentially methylated. **H** Focusing on the promoter regions of the function-annotated genes showed that there were 358 differentially methylated genes at the folding threshold of |log2[*Gm364*-KO/WT]|>2; among them, 79% (284 of 358) were hypermethylated, and 21% (74 of 358) were hypomethylated. **I** KEGG analysis showed that the top three groups of genes were involved in signal transduction, cancers, and immunity and infections. *Indicates *p* < 0.05.

We next investigated how *Gm364* knockout affected transcription. RNA sequencing of WT and *Gm364*-KO ovaries showed that many genes, including both function-annotated and function-unannotated genes, were differentially expressed (Fig. 8A). Of the annotated genes, we found 225 differentially expressed genes (DEGs) at the folding threshold of |log2[*Gm364*-KO/WT]|>2. Among the DEGs, 51% (115/225) were upregulated and 49% (110/225) were downregulated (Fig. 8B, Supplementary dataset 2). KEGG analysis showed that the top three groups of genes were involved in signal transduction, endocrine, and immunity and infections (Fig. 8C). The top up-regulated (Fig. 8D) and down-regulated (Fig. 8E) DEGs were further verified using RT-PCR. Of note, a number of the DEGs, such as *Areg*, *Lrp8*, *Spp1*, and *Cyp19a1*, were reported to be AKT-regulated genes (Fig. 8F, Supplementary Fig. 10), further supporting our working model.

It is known that Notch [13, 14] and Akt [15, 16] are involved in epigenetic regulation in diverse cellular processes, and we therefore next investigated the effect of *Gm364* knockout on the epigenetic status of the ovaries. RRBS of PND 21 ovaries showed that, including CpG, CHG, and CHH methylation, the promoter regions of many genes were differentially methylated (Fig. 8G, Supplementary dataset 3). Of the function-annotated genes, there were 358 differentially methylated genes (DMRs) at the folding threshold of|log2[*Gm364*-KO/WT]|>2. Among these genes, 79% (284/358) were hypermethylated, and 21% (74/358) were hypomethylated (Fig. 8H). KEGG analysis showed that the top three groups of genes were involved in signal transduction, cancers, and immunity and infections (Fig. 8I). KEGG pathways for DEGs and DMRs were largely similar (Fig. 8C, I, Supplementary dataset 4), although we did not find overlap between DEGs and DMRs.

## Discussion

In this study, we show for the first time that an integral transmembrane protein, Gm364, functions to coordinate Notch2 receptor signaling. We also found that active NICD2 can directly phosphorylate (activate) AKT within the cytoplasm to promote meiosis and improve oocyte quality. There are only a few studies about the function of membrane proteins during female meiosis. Nevertheless, compared with these studies, the functional mechanism of Gm364 is distinct. For example, although membrane proteins PNMA5, PLAC1, and FAM70A were all screened as “female fertility factor” together with Gm364 in the same study [8] and all affect p-AKT [3–5], detailed functional studies showed that they worked through distinct pathways from Gm364: PNMA5 functions through SRC-ERK1/2-PNMA5 → AKT [3]; PLAC1 functions through PLAC1-furin-IGF1R-AKT pathway [4]; FAM70A functions through FAM70A-WNT5A-AKT pathway [5]. These suggested the uniqueness of Gm364’s functional mechanism in female meiosis.

Notch signaling is important for follicle formation, survival and development. For example, suppression of Notch signaling abolishes or reduces RA-stimulated expression of STRA8, DAZL, DMC1, and REC8. Notch inhibition greatly impedes the progression of oocytes through meiosis I and impairs primordial follicle assembly in cultured ovarian tissues [17]. Complementary expression of the ligand Jagged1 and its receptor Notch2 in pre-granulosa cells promotes primordial follicle assembly. *Jagged1* knockout within germ cells or *Notch2* knockout within granulosa cells perturbs the primordial follicle pool and antral follicle development [18]. In the present study, we have identified that Gm364 is a key protein involved in follicle survival and development, and functions by maintaining normal Notch2 signaling. We have also identified that Notch2 is the primary Notch member within oocytes.

In our study, we have shown that both oocytes from *Gm364*-KO mice and IVM oocytes treated with Gm364 siRNA both significantly inactivated MIB2/DLL3-Notch2-AKT signaling, suggesting that Gm364 participates in an autocrine signal pathway. We have demonstrated that several oocyte-abundant membrane proteins regulate oocyte meiosis in IVM oocytes, supporting the idea that membrane-generated signals are also essential for oocyte maturation independent of granular cells [3–5]. And several other references also supported this. For example, adenosine present in mouse follicular fluid can prevent oocyte maturation in vitro. The mechanism was that adenosine function on the oocyte membrane to inhibit maturation instead of being transported into oocytes to induce ATP increment [19]. Calreticulin, a well-known endoplasmic reticulum chaperone, is also present on the outer surface of the mouse oocyte membrane. Calreticulin inhibition disrupts cortical actin distribution and meiosis resumption independently of intercellular calcium, indicating that calreticulin could regulate membrane-initiated signaling to resume meiosis [20].

The primary known mechanism of Notch signaling is the cleavage of activated Notch receptors to release NICD, which functions as a transcription factor. However, transcription is quiescent in oocytes, suggesting that NICD2 can function in the cytoplasm to affect meiosis at either the translational or post-translational level. In the present study, we show that *Gm364* knockout reduced p-AKT levels in vivo and that NICD2 could phosphorylate AKT in vitro. This suggests that NICD2 can act as a kinase. NICD2 consists of six ANK motifs. Interestingly, several proteins contain multiple ANK motifs and function upstream to trigger a downstream signaling response [21, 22]. Therefore, based on structurally similar proteins and our in vitro and in vivo data, it is possible that NICD2 also exhibits kinase activity. We also show that AKT might be a key target of NICD2. It is well known that, as the kinase upstream of mTOR, AKT is important for follicle assembly, survival, and activation, and is therefore important for female fertility [11, 12].

**Fig. 9:**
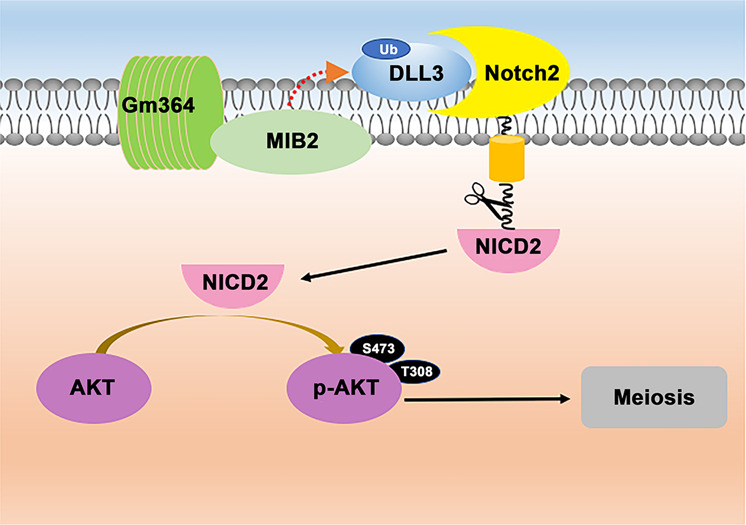
Gm364 working model. On the oocyte membrane, Notch2 and its interactors might transduce signals from outside into the cytoplasm. Gm364 might be a novel essential component of the Notch-containing upstream signal launcher. Gm364 is essential for the membrane enrichment of MIB2, while MIB2 is a ubiquitin ligase required for DLL3 ubiquitination. Next, ubiquitinated DLL3, the active form of DLL3, binds and activates Notch2. Then, the activated Notch2 is cleaved within the cytoplasm to produce NICD2, the intracellular active domain of Notch2. Finally, NICD2 can directly activate AKT within the cytoplasm to regulate oocyte meiosis and quality.

Overall, we have proven in this study that Gm364 is important for Notch2 activation by regulating the correct localization of MIB2 and the normal ubiquitination of the DLL3 ligand. Moreover, we found that NICD2 can directly activate AKT within the cytoplasm to regulate oocyte meiosis and follicle survival and activation (Fig. 9). However, from the wide impacts of *Gm364* knockout on the transcriptional and DNA methylational profiles, the mechanism we explored here might be just partial. Further investigation is needed to find additional new mechanisms on how Gm364 functions at the transcriptional, translational, and post-translational levels. Particularly, close attention might be paid to how Gm364 regulates the levels and activities of the top 10 or 20 DEGs, DEPs (differentially expressed protein), DMRs, and protein PTMs (post-translational modification). This study might contribute to a greater understanding of the roles of integral membrane proteins, as well as Notch2 signaling, in female reproduction.

## Materials and methods

### Animal models

For the global *Gm364*-knockout C57/B6 mice, CRISPR/Cas9 technology was used. We designed two 20-base, gene-complementary oligos of the sgRNA within exon 1 of Gm364 (Supplementary Table 2.1). Each oligo was inserted into pUC57-T7-gRNA. The sgRNA was produced by MEGAshortscript™ Kit (Thermo Fisher Scientific, Waltham, MA, USA) using linearized pUC57-T7-Bin2 gRNA as a template and purified by MEGAclear™ Kit (Thermo Fisher Scientific). The cas9 mRNA was first produced by mMessage mMachine T7 kit (Thermo Fisher) using linearized pST1374-N-NLS-flag-linker-Cas9 as a template. Then the mRNA was poly A-tailed (to increase mRNA stability) using poly A-tailing kit (Thermo Fisher) and purified by RNeasy Micro kit (Qiagen, Dusseldorf, Germany). SgRNA and cas9 mRNA were sent to ACF of SKLSCRB; pronuclear microinjection, embryo transfer, and mouse parturition were accomplished by professional staff in the ACF. Genotyping of *Gm364*-KO mice (Fig. 1E) was done by PCR and DNA sequencing. The forward primer is 5′–GATTCTAAGCTGCTTCCACATTAC–3′; the reverse primer is 5′–TGGCTGGTCTCAGATTTATGAC–3′. The genotyping PCR program was as follows: 94 °C for 5 min, 35 cycles of melting at 94 °C for 30 s, annealing at 57 °C for 15 s, and extension at 72 °C for 30 s, with additional extension at 72 °C for 5 min in the end.

For all fertility assays, 7 WT and 7 *Gm364*-KO B6 female mice were used. WT mating male mice were monthly rotated between cages according to a random allocation table (Supplementary dataset 1), mating started with 2-month-old *Gm364*-KO or WT female mice and WT male mice (all are C57/B6 stains).

Animal experimental procedures in our study were all approved by the Animal Ethics Committee (AEC) of Nanjing Medical University (NJMU), and all mice were housed under standard specific pathogen-free (SPF) conditions of ACF (Animal Core Facility). For the acquisition of uteri or ovaries, mice were first anesthetized with CO_2_, then sacrificed by cervical dislocation.

### Antibodies

Primary antibodies: Mouse monoclonal anti-GAPDH (Cat#: 30201ES60; YEASEN, Shanghai, China); mouse monoclonal anti-β-Actin (Cat#: A5316-100; Sigma, MS, USA); Mouse monoclonal anti-β-Tubulin (Cat#: sc-5274; Santa Cruz, TX, USA); Mouse monoclonal anti‐alpha Tubulin (Acetyl Lys40) (cat#:bsm‐33235M; Bioss, Beijing, China); Human anti-centromere CREST antibody (cat#: 15-234; Antibodies Incorporated, USA); rabbit polyclonal anti-Notch2 (cat#: ab8926, Abcam, Cambridge, UK); Rabbit polyclonal anti-cleaved Notch2 (Asp 1733) antibody (cat#:AF5255; Affinity biosciences, OH, USA); Anti-DLL3 rabbit polyclonal antibody (cat#: Ab103102; Abcam, Cambridge, UK); Anti-MIB2 rabbit polyclonal antibody (cat#: A17829; ABclonal, MA, USA); anti-Phosphorylation RICTOR (Thr1135) (cat#: D30A3, Cell Signaling Technology, MA, USA); Anti-AKT (Ab-129) Rabbit polyclonal antibody (Cat#: D151616-0100; BBI Life science, Shanghai, China); Rabbit anti-Phospho AKT (Thr308, Cat#: 13038, Cell Signaling Technology); Rabbit anti-Phosphpho AKT (Ser473, Cat# 4060, Cell Signaling Technology); Mouse monoclonal anti-strep II Tag (Cat#: YFMA0054, Yifeixue, Nanjing, China); mouse monoclonal anti-flag Tag (Cat#: D190828, BBI Life science). Anti-Gm364 rabbit polyclonal antibody was made by Yingji BioTech (Rabbit #: E1171; Shanghai, China) and purified by affinity purification. The antigen is 1-208 AA of mouse Gm364 protein (NP_001122097.1).

Secondary antibodies: Horseradish peroxidase (HRP)-conjugated rabbit anti-goat IgG and HRP-conjugated goat anti-mouse IgG were purchased from Vazyme (Nanjing, Jiangsu, China). Cy2-conjugated donkey anti-rabbit IgG (Code: 711-225-152), Cy2-conjugated donkey anti-mouse IgG (Code: I715-225-150), and Rhodamine(TRITC)-conjugated donkey anti-rabbit IgG (Code: 711-025-152) were purchased from Jackson ImmunoResearch Laboratory (West Grove, PA, USA).

### Oocyte collection and in vitro culture

Fully-grown GV oocytes were collected from three-week-old female mice. Oocytes were released by puncturing follicles with a sterile syringe needle in a MEM + medium (0.01 mM EDTA, 0.23 mM Na-pyruvate, 0.2 mM penicillin/streptomycin, and 3 mg/ml BSA in MEM). After washing away the cumulus cells from the cumulus-oocyte complexes, every 50 oocytes were cultured in 100 µl mini-drops of MEM + containing 20% fetal bovine serum (FBS) (Thermo Fisher) covered with mineral oil at 37.0 °C in an incubator with 5% O_2_, 5% CO_2_, and a humidified atmosphere.

For y-secretase inhibition, RO4929097 (Selleck) was added into MEM+ at 4 nM and oocytes were treated for 24 h. For AKT inhibition, perifosine (Selleck) was added into MEM+ at 3 μM and oocytes were treated for 16 h.

### In vitro fertilization (IVF)

Spermatozoa were obtained from the epididymis of 10–18 weeks old B6-DBA2 F1 male mice and were then capacitated in 1 ml MEM+ for 1 h. Subsequently, 10 µl of the suspension containing 5–10 × 10^6^/ml spermatozoa was added to 490 µl MEM+ medium, and γ-secretase-inhibited (γ-secretase (-)) oocytes were added. Five hours later, the sperms remaining on the surface of oocytes were washed off by pipetting. After another 4 h, the oocytes were processed for immunostaining to determine the frequency of successful fertilization, by the identification of the formation of pronuclei.

### In vitro culture of in vivo fertilized oocytes from 2-cell to blastula

5 IU PMSG were first injected intra-peritonelly into 6-week-old WT or Gm364-KO B6 female mice. 46 h later, 5 IU HCG were injected intra-peritonelly and mated with 10-week-old WT B6 male mice. Twelve to fourteen hours later, the female mice with visible vaginal plugs (which indicates successful fertilization) were anesthetized and sacrificed, and fertilized zygotes were obtained from the tubal ampulla and transferred into MEM+ for 2-cell development. After the 2-cell stage, the cells were transferred into KSOM (Cat no: MR-020P-5F, Millipore, MA, USA) for the development from 4-cell to blastula.

### Immunofluorescence staining of oocytes

Fifty oocytes per repeat were permeabilized with 0.5% Triton X-100/PHEM (60 mM PIPES, 25 mM Hepes, pH 6.9, 10 mM EGTA, 8 mM MgSO_4_) for 5 min, and then were fixed in 3.7% FPA in PHEM for 20 min at room temperature. After being washed with PBS/0.05% PVP (polyvinylpyrrolidone) three times at 10 min each, oocytes were blocked in blocking buffer (100 mM glycine and 1% BSA in PBS) for 1 h at room temperature. Primary antibodies were then diluted in blocking buffer and oocytes were incubated in it overnight at 4.0 °C. The following steps were performed as described above.

### Ovarian hematoxylin-eosin staining and ovary follicle counting

2-month-old WT and *Gm364*-KO female mice were used for follicle counting. To eliminate the effects of distinct estrous cycles among different mice, 10 IU/mice PMSG were intraperitoneally injected into mice to synchronize the estrous cycle. Ovaries were obtained after 48 h, washed, and fixed in 10% buffered formalin or 4% PFA overnight, embedded in paraffin, continuously sectioned at 5 µm thickness, then stained with hematoxylin and eosin. The follicle stages were classified according to Pederson’s standard. In brief, an oocyte surrounded by a single layer of flattened or cubical granulosa cell was defined as a primordial or primary follicle; an oocyte surrounded by more than one layer of cuboidal granulosa cells without visible antrum was defined as a secondary follicle; a follicle possessing a clearly antral space containing follicular fluid was defined as an antral follicle. For the follicle number, only follicles with visible nuclei were counted. And we counted every other two slices (since the same follicle will appear at adjacent different slices) and the final follicle number of a certain stage is a cumulative number of all counts of the corresponding stage. The number of follicles counted per ovary was used for statistical analysis.

### Lipid raft extraction

UltraRIPA kit for Lipid Raft (BioDynamics Laboratory Inc.) was used to extract lipid raft protein. Two-month-old ICR female mice’ ovary was collected to a clean tube with 200 μl A-buffer and homogenized with a sonicator. Next, the lysed sample was centrifuged and the supernatant is RIPA-soluble fraction (Sol in supplementary figure 3). Next, the pellet was resuspended in A-buffer, then B-buffer was added, mixed, and incubated at room temperature for 5 min. Finally, the mixture was centrifuged and the supernatant is lipid raft fraction (Insol in Supplementary Fig. 3). The successful extraction of both soluble and insoluble fractions was verified by the presence of ERK in both fractions (Supplementary Fig. 3).

### Embryo staining and counting

About 500 μl of 0.4% trypan blue (Cat No. 72-57-1, Solarbio Life Sciences, Beijing, China) was injected into the pregnant mice via the caudal vein. After 0.5 h, the mice were sacrificed, the uteri were obtained, and the site or embryo with clearly delineated signal was counted as an implantation site (DPC 5) or implanted embryo (DPC 10).

### Immunoprecipitaion

In total, 2.5 μg rabbit anti-Gm364 antibody, rabbit anti-Notch2 antibody, rabbit anti-NICD2 antibody, rabbit anti-MIB2 antibody, rabbit anti-DLL3 antibody, rabbit anti-AKT antibody, or rabbit anti-RICTOR antibody was first coupled to 30 μl protein A/G beads (Yeasen, Shanghai, China) for 4 h at 4 °C on a rotating wheel in 250 μl immunoprecipitate (IP) buffer (20 mM Tris-HCl, pH 8.0, 10 mM EDTA, 1 mM EGTA, 150 mM NaCl, 0.05% Triton X-100, 0.05% Nonidet P-40, 1 mM phenylmethylsulfonyl fluoride) with 1:100 protease inhibitor (Sigma) and 1:500 phosphatase inhibitor (Sigma). Meanwhile, 400 oocytes were lysed and ultrasonicated in 250 µl IP buffer and then pre-cleaned with 30 µl protein-A/G beads for 4 h at 4 °C. After that, protein A/G-coupled Rabbit IgG or specific antibodies were incubated overnight at 4 °C with pre-cleaned oocyte lysate supernatant. Finally, after being washed three times (10 min each with 250 µl IP buffer), the resulting beads with bound immunocomplexes were subjected to SDS-PAGE and western blot.

### Silver staining and characterization of Gm364-interacting proteins

For silver staining, immunocomplex beads (obtained as in immunoprecipitation) from the control IgG or Gm364 antibody group were boiled in protein sample buffer, and supernatants were separated side by side on an SDS-PAGE gel. The gel was firstly fixed overnight in 10% acetic acid and 40% ethanol and sensitized for 30 min at room temperature with a fresh sensitizing solution (30% ethanol, 0.2% Na_2_S_2_O_3_, 0.314% Na_2_S_2_O∙5H_2_O and 6.8% sodium acetate). Following being washed three times with water for 5 min each, the gel was then stained for 20 min at room temperature in staining solution (0.25% AgNO_3_, 0.02% of fresh 37% formaldehyde solution), washed with water for 2.5 min, and developed for about 5–10 min in developing solution (2.5% NaCO_3_, 0.02% of fresh 37% formaldehyde solution). Finally, 0.4% glycine was used to stop the developing reaction.

To identify Gm364-interacting proteins, silver-stained control and Gm364 lanes were compared carefully, and those bands with the remarkably higher gray level in the Gm364 lane were cut out one by one and stored in protease-free tubes with 10% ethanol. The selected bands, which were potentially Gm364 integrators, were then sent to the Testing and Analysis Center (Nanjing Medical University) to undergo matrix-assisted laser desorption/ionization-time of flight mass spectrometry. The identity of each protein was given in peptide mass fingerprinting searches in Mascot (http://www.matrixscience.com/mascot/cgi/search_form.pl?FORMVER=2&SEARCH=PMF).

### Expression and purification of recombinant NICD2 and AKT proteins with bac-to-bac system

EGFP-strep II-tagged NICD2, TagRFP-flag-tagged AKT proteins were cloned and expressed through a Bac-to-bac system (Thermo Fisher). In brief, the corresponding sequences (Supplementary Table 1) were cloned into pFastBacHTA (Thermo Fisher) and then transformed into DH10Bac *E. coli* cells. The bacmid was isolated from DH10Bac E. coli (Vazyme) with QIAfilter Plasmid Purification Kit (Qiagen) and then transfected into Sf9 cells with Cellfectin® II Transfection reagent (Thermo Fisher) in the static upper plane of an orbital shaker (Shanghai Zhichu Instrument Co. Shanghai, China) at 27 °C. The virus-containing supernatant was used to infect fresh Sf9 cells for 72 h, and the procedure was repeated twice to amplify the baculovirus. In total, 20 μl of the third cycle supernatant was added to Sf9 cells in 250 ml SFM900-II medium (Thermo Fisher) with 5% FBS to express proteins in the lower bed of an orbital shaker at 27 °C, 200 rpm. Next, infected cells were resuspended in a lysis buffer (containing 50 mM Tris, 10% sucrose, 50 µM ATP, 1 mM PMSF, 5 mM DTT, 1% NP40, 10 mM imidazole, 1× protease inhibitor and phosphatase inhibitor, PH 7.0 by HCl) and lysed with a high-pressure cell disrupter (Union Biotech, Shanghai, China), centrifuged, and the resulting supernatant was incubated with 1 ml Ni-NTA Superflow resin (Qiagen) at 4 °C for 1 h. The resin was then transferred into a 5 ml chromatography column (Biocomma Co., Shenzhen, Guangdong, China), washed with four column volumes of wash buffer (40 mM imidazole in resuspension buffer without PMSF), and eluted with 500 mM imidazole in resuspension buffer without PMSF. The eluted protein was concentrated by a size-exclusion spin column and exchanged into BRB80 (80 mM HEPES, 1 mM MgCl_2_, 1 mM EGTA, pH 6.8 by KOH) with 10% glycerol, 50 μM ATP, and 5 mM DTT. The protein was aliquoted and kept in a −80° freezer for future use.

### Expression and purification of recombinant RPS6 proteins in *E. coli*

Recombined RPS6-tagRFP-flag sequence was cloned into pRSETB (primers are in supplementary table 1) and transformed into BL21-DE competent *E. coli* (Vazyme). The transformed *E. coli* was grown in 4 × 1 L LB + medium in an orbital shaker (Shanghai Zhichu Instrument Co.) at 37 °C, 220 rpm until OD600 reached 1.0. Next, 0.2 mM IPTG was added into the medium to induce the recombined RPS6 expression overnight at 16 °C, 220 rpm. The next morning, the *E. coli* was spun down, washed once with cold PBS, lysed, and purified as described above.

### In vitro phosphorylation assays

Each two of NICD2-strep II, AKT-flag, and RPS6-flag proteins were mixed at equal molar ratio in BRB80 with 10% glycerol, 1 mM ATP, and 5 mM DTT and incubated at RT for 20 min. Finally, the reaction was subjected to a western blot.

### Assay of mitochondrial transmembrane potential

Fifty oocytes per repeat were incubated at 37 °C for 20 min with the fluorescent potentiometric indicator JC-1 (Cat#: 40706ES60, Yeasen, Shanghai, China) diluted 1:200, then washed twice with PBS and added to droplets (50 µl) of culture medium. Images of green fluorescence (JC-1 as monomers at low membrane potentials) and red fluorescence (JC-1 as aggregates at higher membrane potentials) were captured using confocal microscopy as above. Mitochondrial depolarization is indicated by a decrease in the red/green fluorescence intensity ratio.

### Detection of ROS generation

The ROS Assay Kit (Cat#: S0033, Beyotime, Beijing, China) was used to detect ROS generation in oocytes. In brief, 50–100 oocytes per repeat were incubated with a dichlorofluorescein diacetate (DCFH-DA) probe for 20 min at 37 °C in the dark, washed, and mounted on slides for confocal imaging.

### Immunogold-electron microscopy of oocytes

For MIB2/DLL3 or MIB2/Gm364 co-labeling and signal acquisition through Immunogold-electron microscopy, 50–100 oocytes per repeat were blocked with 1% BSA, incubated with MIB2 Ab at 4 °C overnight, next labeled with Donkey anti-rabbit IgG/15 nm-Gold at room temperature for 2 h. Then oocytes were re-blocked with 1% BSA (to eliminate the cross-reaction between two antibodies), incubated with DLL3 or Gm364 Ab (4 °C, overnight), and then labeled with Donkey anti-rabbit IgG/35 nm-Gold or 10 nm-Gold at room temperature for 2 h.

After being labeled, oocytes were fixed in 2.5% glutaraldehyde for 2 h at 4 °C, washed three times with PBS, and stained with eosin for 2 min to facilitate the oocyte positioning by eye. Oocytes were then placed in 2% agarose and spun for 5 min at 13,000 rpm and held overnight at 4 °C. The next day, the agarose piece with eosin-stained oocytes (red in the piece) was trimmed and sent to the Testing and Analysis Center (Nanjing Medical University) for sample preparation for transmission electron microscopy. Electron microscopy pictures were obtained with a transmission electron microscope (FEI Tecnai G2 Spirit Bio Twin; Thermo Fisher Scientific).

### Chromosome spread and aneuploidy appraisal

50 oocytes were exposed to Tyrode’s buffer (pH 2.5) for 40–50 s to remove the zona pellucida, then fixed in a drop of 1% paraformaldehyde with 0.15% Triton X-100 on a glass slide. Kinetochore and chromosome were then stained for judgment of chromosome pairs, If the chromosome pair number of an oocyte is not 20 (the chromosome pair number of a normal euploidy cell in mouse), then it is an oocyte with aneuploidy. The aneuploidy percentage is the number of oocytes with aneuploidy divided by the number of total examined oocytes.

### RNA sequencing and analysis

RNA samples were collected from mouse ovaries. RNA isolation, library construction, and RNA sequencing (RNA-seq) were carried out by the Beijing Genomics Institute following standard protocols. The library products were sequenced using a BGISEQ-500. Standard bioinformatics analysis was performed by the Beijing Genomics Institute. For gene expression analysis, the significance of the differential expression genes was defined by the bioinformatics service of BGI according to the combination of the absolute value of|log2(treated/control)|≥ 2 and *q* value <0.001. All original sequence datasets have been submitted to the database of NCBI Sequence Read Archive (SRA) under accession number.

### Reduced representation bisulfite sequencing (RRBS)

Frozen ovaries were briefly ground in lysis buffer, and then, DNA was extracted with QIAamp DNA Blood Maxi Kit (Qiagen) according to the manufacturer’s recommendation. Reduced representation bisulfite sequencing was performed by Shanghai BioGenius Biotech Inc as previously described. In short, DNA samples were bisulfite-treated with an EZ DNA Methylation-Gold kit (ZYMO Research) to create the library. Sequencing reads were converted and loaded on Illumina HiSeq 3000 platform. Differentially methylated regions analysis was performed by swDMR software.

### Quantification of ROS, JC-1, immunofluorescence, western blot, agarose gel, and γ-H2AX images

We always used original tif images for quantification of ROS, JC-1, immunofluorescence, western blot, or agarose gel images. The image is opened in image j (National Institutes of Health, Bethesda, MD, USA), For intensity measurement in ROS, JC-1, and immunofluorescence, signal intensity was first measured, then background intensity was also measured, the net intensity was obtained from signal intensity subtracted by background intensity. For gross intensity measurement in a western blot or agarose gel, the net intensity was obtained as above, then the band area was measured, finally, the integrated intensity was obtained from net intensity multiplied by band area.

For γ-H2AX signal quantification, we count the “signal dot” as most researchers did. For accurate and efficient counting, an original tif image was magnified and the signal region was subdivided into several small regions. Counts from all small regions were finally added together.

### siRNA-mediated Gm364 knockdown in oocytes

*Gm364* DNA templates for small interfering RNA (siRNA) were in supplementary table 2. siRNAs were produced and purified by using the T7 Ribomax Express RNAi System (Promega, Madison, WI, USA) according to the manufacturer’s instructions. Purified siRNAs were aliquoted and stored at −80 °C after a quality check on an agarose gel. A ready-to-use siRNA mixture was set by mixing siRNAs against four distinct target regions at an equal molar ratio to a final concentration of 5 µM.

For siRNA-mediated Gm364 knockdown in oocytes, we used the N-TER nanoparticle siRNA transfection system (Millipore, Sigma) and operated as reported [4, 5]. During the entire siRNA treatment, typically 36–44 h long, 2.5 mM milrinone was included to prevent the resumption of meiosis. Oocytes were then collected for experiments in supplementary figure 6.

### Animal/individual sample inclusion, experiment grouping, data collection, and data analysis

Any selected oocyte has to be of normal quality (fully-grown oocyte from an antral follicle, normal diameter, tight connection between zona pellucida and oocyte membrane, etc.). Any selected female mouse has to be physically healthy (normal body weight, eats normally, normal activity, etc.). Any oocyte or mouse that is of bad quality or unhealthy will be excluded.

For all experiment grouping, data collection, and data analysis, we tried to follow the blinding roles. Data collection, data analysis, and data inputting (into excel files) were accomplished by different authors.

For experiment grouping and data collection in fertility assays (Figs. 1I and J), each mating cage was assigned a unique cage number without being labeled with “WT” or “*Gm364*-KO”. Every day one author examined each cage and documented newborns, then the data were sent to one first author (one of the five first authors). The first author inputted the daily data into the fertility assay excel file, where each cage number corresponds to a specific WT or *Gm364*-KO female mice.

For all other experiment groupings and data collection, control or treated (*Gm364*-KO or Gm364-knockdown) samples have to be clearly labeled. However, during the image taking, follicle counting, or intensity quantification, the group label for each sample was covered by a black sticky tap and relabeled as numbers or letters. After the processes, the black sticky taps were removed, one first author could easily find the correlation between the analyzed data and the sample information and inputted the data into the corresponding original excel file.

Before the experiment operation, each individual (oocyte, ovary, or mice) in an independent repeat or group was selected, allocated randomly and blindingly. For data collection in an independent repeat, each data point was selected randomly.

### Statistical analysis

All statistical graphs for western blots or DNA gels were from three independent repeats, and all statistical graphs for blood biochemical indexes were from five independent repeats. The sample size for all other graphs was indicated as “*n* = *x*” above the graphs. If the standard error of all randomly collected individual data points in a group is significantly smaller than the average value, the corresponding sample size is appropriate and creditable. Data are presented as mean ± SEM. Statistical comparisons between two groups were made with the Student’s *t*-test of the Excel program (Microsoft, Redmond, WA, USA). Multiple comparisons were made by using the Kruskal–Wallis one-way nonparametric ANOVA (Prism; GraphPad Software, San Diego, CA, USA). Values of *P* < 0.05 were considered statistically significant.

## Supplementary information

Supplementary tables

Supplementary legends

Supplementary Figure 1

Supplementary Figure 2

Supplementary Figure 3

Supplementary Figure 4

Supplementary Figure 5

Supplementary Figure 6

Supplementary Figure 7

Supplementary Figure 8

Supplementary Figure 9

Supplementary Figure 10

Supplementary dataset 1

Supplementary dataset 2

Supplementary dataset 3

Supplementary dataset 4

## Data Availability

The detailed procedures of methods, nine figures, ten supplementary figures, two supplementary tables, and four supplementary datasets are attached. Supplementary datasets 1-4 have also been deposited into Zenodo (https://zenodo.org/deposit/5035429), the DOI is 10.5281/zenodo.5035429.
