# Supplementary material for: Gm364 coordinates MIB2/DLL3/Notch2 to regulate female fertility through AKT activation
Source: Cell Death Differ. 2021 Oct 11;29(2):366–80. doi: 10.1038/s41418-021-00861-5 (PMC8816931; doi:10.1038/s41418-021-00861-5)
Supplement: Supplementary file 1 — Supplementary tables [file 41418_2021_861_MOESM1_ESM.doc]

**Supplementary table 1. DNA templates for RT-PCR and plasmid construction**

| **Primer** | **DNA templates** |
| --- | --- |
| Notch1-RT-F | GCATTGATGATGTCGCTGG |
| Notch1-RT_R | CACACTCATTGATGTCAACCTCG |
| Notch2-RT-F | GTGTTCAGGAAAAGGCAAGG |
| Notch2-RT-R | CCGATGAAGTCATTGCAGG |
| Notch3-RT-F | AGTTTACTTGCATCTGCATGG |
| Notch3-RT-R | CGACACTCATAGCCGTCAG |
| Notch4-RT-F | CACCTGTCACCAAGATCTGG |
| Notch4-RT-R | TTGACCTCCACCTCACAGAG |
| DLL1-RT-F | GAAGGAAGCCACTGCAAGC |
| DLL1-RT-R | TCTCGGCTCCAGGTGTGC |
| DLL3-RT-F | TGCACTCAACAACCTGAGGTTAC |
| DLL3-RT-R | CAGTCGGTAGGGGGAGGTAGAG |
| DLL4-RT-F | AGCCAAAGTCACTTGGGTGC |
| DLL4-RT-R | CTGCTTGTTAGGGATGTCGCTC |
| Akt-RT-F | CAGGATGTGGATCAGCGAG |
| Akt-RT-R | CTCATCCTTGGCGACGATG |
| Akt-pFastBac-F | ACGCGTCGACTAGCCACCAACGACGTAGCCATTGTGAAG |
| Akt-pFastBac-R | AAGGAAAAAAGCGGCCGCGGCTGTGCCACTGGCTGAG |
| NICD2-pFastBac-F | CCGGAATTCGTCATCATGGCCAAGCGGAAG |
| NICD2-pFastBac-R | CGCGGATCCCTGCATACACCTGCATGTTGC |
| Rps6-flag-F-pRSETB-F | CCGGAATTCAAGCTGAACATCTCCTTCCCCG |
| Rps6-flag-F-pRSETB-R | CCCAAGCTTTTATTTATCGTCATCGTCTTTATAGTC |

**Supplementary table 2. DNA oligos for Gm364 siRNA**

| **Target Site** | **DNA templates** |
| --- | --- |
| Gm364 CDS 169-193^1^ | Oligo1: GGATCCTAATACGACTCACTATAGAGGGTCATCCCAACTCCTATTGTA^2^ |
|  | Oligo2: AATACAATAGGAGTTGGGATGACCCTCTATAGTGAGTCGTATTAGGATCC^2^ |
|  | Oligo3: GGATCCTAATACGACTCACTATATACAATAGGAGTTGGGATGACCCTC^2^ |
|  | Oligo4: AAGAGGGTCATCCCAACTCCTATTGTATATAGTGAGTCGTATTAGGATCC^2^ |
| Gm364 CDS 1010–1034^1^ | Oligo1: GGATCCTAATACGACTCACTATAGAGACATTGCCAAATTTAACCGTAT^2^ |
|  | Oligo2: AAATACGGTTAAATTTGGCAATGTCTCTATAGTGAGTCGTATTAGGATCC^2^ |
|  | Oligo3: GGATCCTAATACGACTCACTATAATACGGTTAAATTTGGCAATGTCTC^2^ |
|  | Oligo4: AA GAGACATTGCCAAATTTAACCGTATTATAGTGAGTCGTATTAGGATCC^2^ |
| Gm364 CDS 1335–1359^1^ | Oligo1: GGATCCTAATACGACTCACTATAGACAACAGTGTTATTTCCTGGAATT^2^ |
|  | Oligo2: AAAATTCCAGGAAATAACACTGTTGTCTATAGTGAGTCGTATTAGGATCC^2^ |
|  | Oligo3: GGATCCTAATACGACTCACTATAAATTCCAGGAAATAACACTGTTGTC^2^ |
|  | Oligo4: AAGACAACAGTGTTATTTCCTGGAATTTATAGTGAGTCGTATTAGGATCC^2^ |
| Gm364 CDS 1735–1757^1^ | Oligo1: GGATCCTAATACGACTCACTATAGAAGTTACAGTTCTGCTATGCTA^2^ |
|  | Oligo2: AATAGCATAGCAGAACTGTAACTTCTATAGTGAGTCGTATTAGGATCC^2^ |
|  | Oligo3: GGATCCTAATACGACTCACTATATAGCATAGCAGAACTGTAACTTC^2^ |
|  | Oligo4: AAGAAGTTACAGTTCTGCTATGCTATATAGTGAGTCGTATTAGGATCC^2^ |
| Control^3^ | Oligo1: GGATCCTAATACGACTCACTATAGACCTACGCCACCAATTTCGT^2^ |
|  | Oligo2: AAACGAAATTGGTGGCGTAGGTCTATAGTGAGTCGTATTAGGATCC^2^ |
|  | Oligo3: GGATCCTAATACGACTCACTATAACGAAATTGGTGGCGTAGGTC^2^ |
|  | Oligo4: AAGACCTACGCCACCAATTTCGTTATAGTGAGTCGTATTAGGATCC ^2^ |
