# Supplementary material for: Gm364 coordinates MIB2/DLL3/Notch2 to regulate female fertility through AKT activation
Source: Cell Death Differ. 2021 Oct 11;29(2):366–80. doi: 10.1038/s41418-021-00861-5 (PMC8816931; doi:10.1038/s41418-021-00861-5)
Supplement: Supplementary file 2 — Supplementary legends [file 41418_2021_861_MOESM2_ESM.doc]

**Supplementary Figure legends**

**Supplementary Figure 1. Genomic sequencing of *Gm364* region in WT and *Gm364*-knockout mice**

A. A sketch map showed that through the Cas9 technique, 55 bp DNA sequences within exon 1 of *Gm364* gene were deleted. B. Sanger sequencing of exon1 of *Gm364* gene in WT and *Gm364*-KO mice.

**Supplementary Figure 2. Verification of Gm364-interacting proteins**

A-D. Co-IP and blots showed that Gm364 interact with TTC37 (B), MIB2 (C), and GRAMD1A (D), which were identified potential Gm364 interactors through IP-MALDI (A). E-H. Blots and quantifications showed that *Gm364* knockout didn't affect the protein levels of GRAMD1A (E and F) and TTC37 (G and H), although they all interact with Gm364. β-Actin was used as a loading control.

**Supplementary Figure 3. Characterization the existence of all Gm364-related proteins within the soluble and insoluble (lipid raft) fraction of oocyte membrane**

Oocytes membrane were extracted and further separated into soluble and insoluble (lipid raft) fractions. Blot showed that ERK was present in both fractions, while Gm364, MIB2, DLL3, NOTCH2, and NICD2 all existed only in the soluble fraction.

**Supplementary Figure 4. *Gm364* knockout significantly reduced p-AKT level.**

A-C. Immunofluorescence showed that *Gm364* knockout significantly reduced p-AKT levels at S473 and T308 while didn't alter total AKT level. Scale bar, 10 μm.

**Supplementary Figure 5. *Gm364* knockout didn't affect the levels of various important meiotic kinases**

A. Immunofluorescence showed that p-AKT^S473^ co-localized with NICD2 in GV oocytes. DNA in blue, p-AKT^S473^ in green, NICD2 in red. B and C. Blot and quantification showed that *Gm364* knockout didn't affect other important meiotic kinases including p-β-Catenin, p-SRC, and p-ERK. Scale bar, 20 μm.

**Supplementary Figure 6. Gm364 knockdown and AKT inhibition both decrease oocyte maturation**

A and B. Blots and quantifications showed that Gm364 was significantly reduced by siRNA. C and D. Immunofluorescence and quantifications showed that Gm364 intensities at membrane and within cytoplasm were both significantly reduced by siRNA. E and F. Gm364 knockdown dramatically decreased oocyte GVBD rate. G. Gm364 knockdown dramatically decreased oocyte MII rate (1pb, first polar body). H and I. Blots and quantifications showed that the levels of NICD2 and p-AKT^S473^ was significantly reduced by Gm364 knockdown. J and K. Blots and quantifications showed that p-AKT^S473^ level was significantly reduced by specific inhibitor perifosine. L and M. AKT inhibition significantly reduced oocyte maturation (1pb rate). Scale bar for C, 20 μm; scale bar for L, 100 μm. * indicates p ˂ 0.05.

**Supplementary Figure 7. *Gm364* knockout didn't alter body & organ weights and major blood biochemical indexes**

A and B. *Gm364* knockout didn't affect the weights of the whole body (A) and major organs (B) of female mice. C-N. Assays of 12 key biochemical indexes showed no significant differences between the WT and *Gm364-*KO groups. From C to N, ALT, BUN, AST, CHOL, CK, CREA, HDL-C, LDH, LDL-C, SOD, TG, and UA.

**Supplementary Figure 8. *Gm364* knockout didn't affect the inflammation and immunity of the liver and kidney.**

A-D. Blot and quantification of the liver (A and B) and kidney (C and D) samples showed that *Gm364* knockout had no obvious effects on the levels of CD4, CRP, IL4, and IL6. α-tubulin was used as a loading control. Scale bar for E and G, 200 μm.

**Supplementary Figure 9. *Gm364* knockout didn't affect the apoptosis and proliferation of the liver and kidney.**

A-D. Blot and quantification of the liver (A and B) and kidney (C and D) samples showed that *Gm364* knockout had no obvious effects on the levels of Bax and Bcl2. α-Tubulin was used as a loading control. E-H. Tissue immunofluorescence and quantification in the liver (E and F) and kidney (G and H) showed that *Gm364* knockout had no obvious effects on the Ki67 level. Scale bar, 200 μm.

**Supplementary Figure 10.** **Interaction between AKT and DEGs *between Gm364-knockout* and WT ovaries.**

AKT interacting networks. Various DEGs (Fig. 8F) between *Gm364*-KO and WT ovaries are regulated by AKT.

**Supplementary table legends**

**Supplementary table 1. DNA templates for RT-PCR and plasmid construction**

**Supplementary table 2. DNA oligos for Gm364 siRNA**

^1^ The numbers are the starting and ending position of the target sites in Gm364 CDS (NM_001128625.2 in NCBI).

^2^ two pairs of DNA oligos are needed for for each double-stand siRNA. Oligo 2 is complementary with oligo 1 except an "AA" overhang at 5'; Oligo 3 is complementary with oligo 4 except an "AA" overhang at 5'. In each oligo, gene-specific sequences are underlined, other sequences are for recognition and binding by T7 RNA polymerase.

^3^ Control siRNA does not target to any mRNA sequence in mouse.

**Supplementary dataset legends**

**Supplementary dataset 1.** Related to fertility assays in Figure 1I and 1J. WT mating male mice were monthly rotated between cages according to this random allocation table. Dates are presumptive.

**Supplementary dataset 2.** Related to figure 8A-C. This excel file contains FPKM values of three WT or *Gm364*-KO repeats, Log2-Ave value, and Log2(Ave-*Gm364*-KO/ave-WT) for each gene. To avoid the illegal calculation of value “0”, we added a minimal value “0.001” to all original values (we have verified that this did not alter any differential trends). The data were arranged to the ascending order of Log 2(Ave-*Gm364*-KO/ave-WT) values.

**Supplementary dataset 3.** Related to figure 8G-I. This excel file contains six sheets. "CPG-all info", "CHG-all info", and "CHH-all info" contain all original information for CPG, CHG, or CHH promoter regions of each gene; "CpG methyl value", "CHG methyl value", and "CHH methyl value" contain methylation values, Ave methylation values of three WT or *Gm364*-KO repeats, Log2-Ave value, and Log2(Ave-*Gm364*-KO/ave-WT) for CPG, CHG, or CHH promoter regions of each gene. To avoid the illegal calculation of value “0”, we added a minimal value “0.001” to all original values (we have verified that this did not alter any differential trends). The data were arranged to the ascending order of Log 2(Ave-*Gm364*-KO/ave-WT) values.

**Supplementary dataset 4.** Related to figure 8A and 8G. This excel file contains two sheets, "RNA seq up & down top 20" and "RRBS up & down top 20" including top 20 up-regulated & down-regulated DEGs and DMRs.
