# Supplementary figures and images for: Gm364 coordinates MIB2/DLL3/Notch2 to regulate female fertility through AKT activation
Source: Cell Death Differ. 2021 Oct 11;29(2):366–80. doi: 10.1038/s41418-021-00861-5 (PMC8816931; doi:10.1038/s41418-021-00861-5)

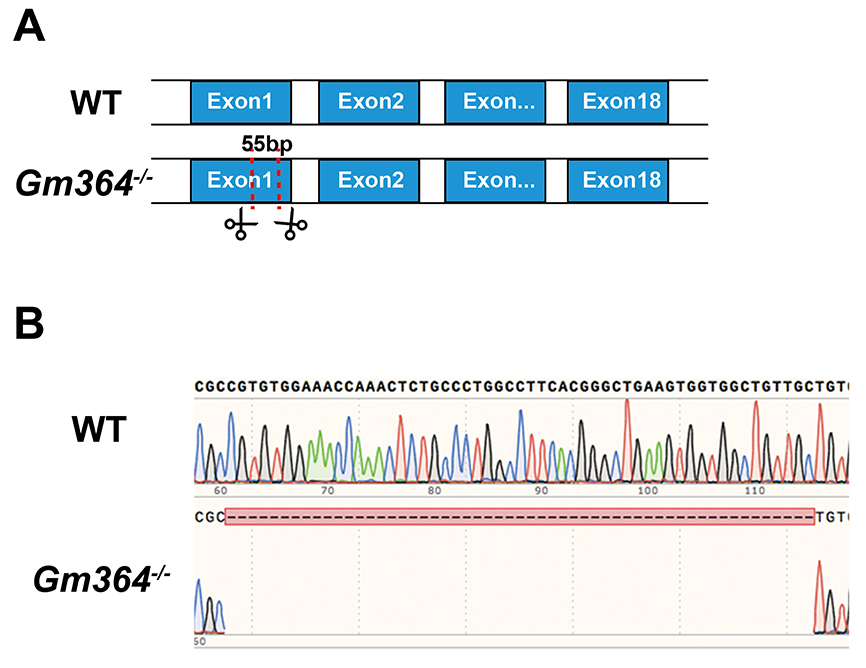

Supplement: Supplementary file 3 — Supplementary Figure 1 [file 41418_2021_861_MOESM3_ESM.tif]

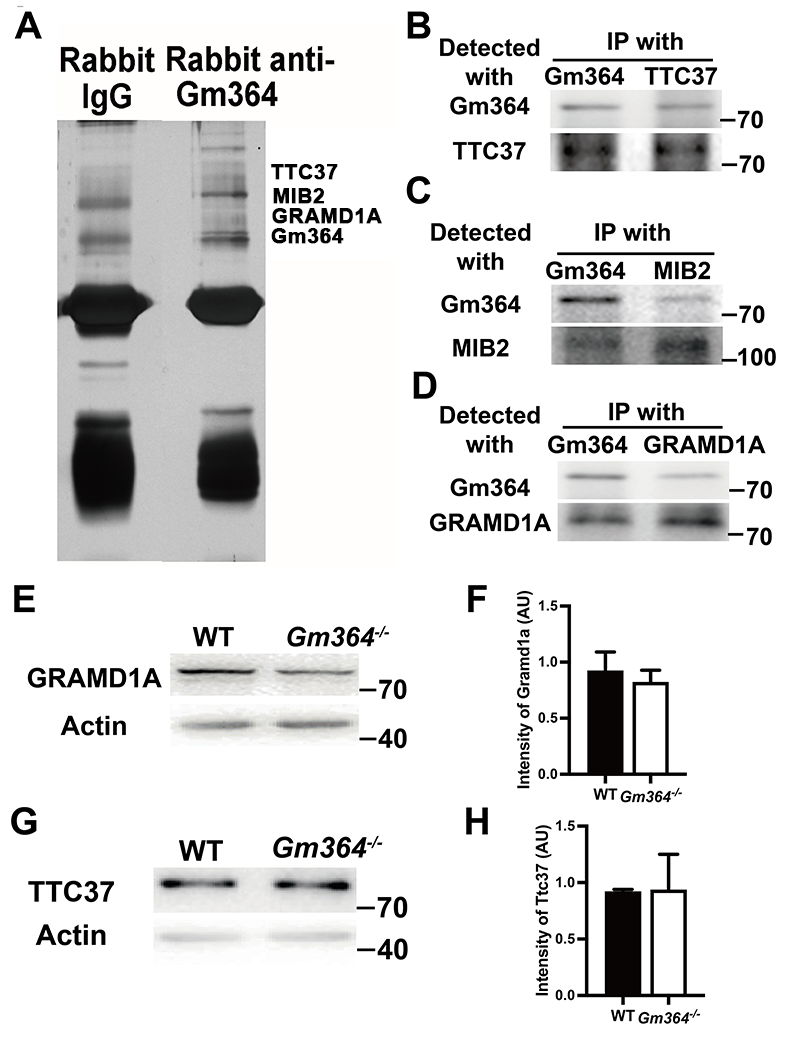

Supplement: Supplementary file 4 — Supplementary Figure 2 [file 41418_2021_861_MOESM4_ESM.tif]

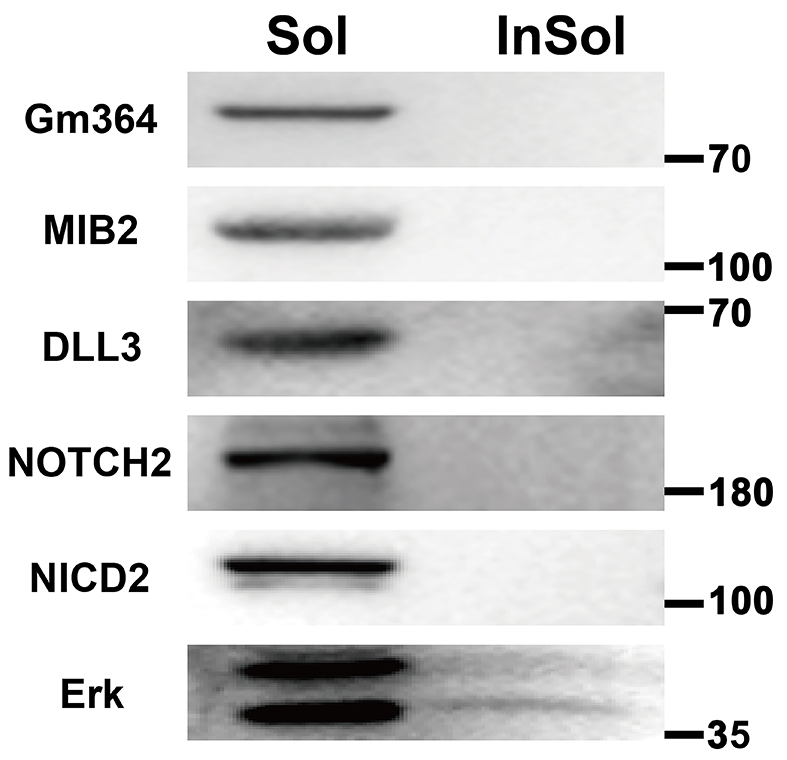

Supplement: Supplementary file 5 — Supplementary Figure 3 [file 41418_2021_861_MOESM5_ESM.tif]

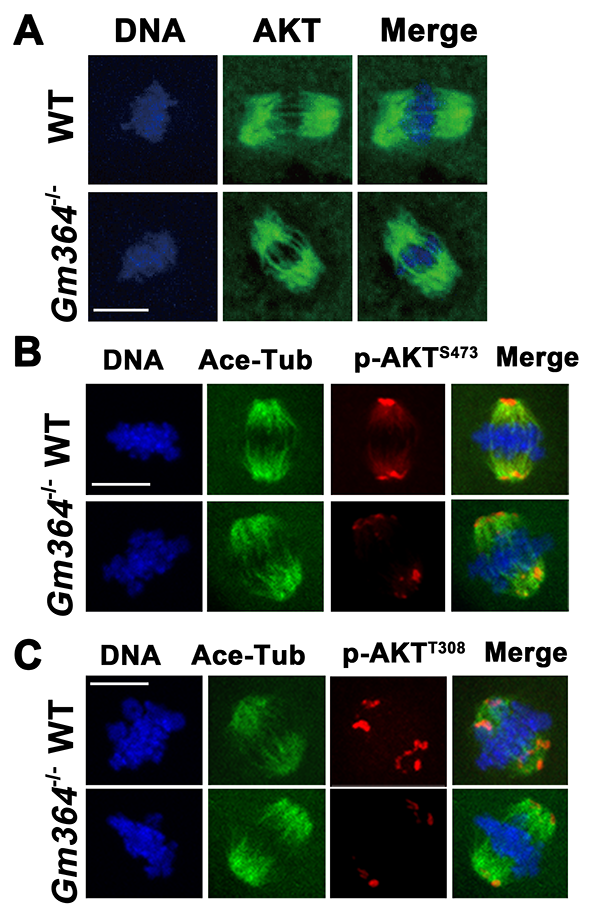

Supplement: Supplementary file 6 — Supplementary Figure 4 [file 41418_2021_861_MOESM6_ESM.tif]

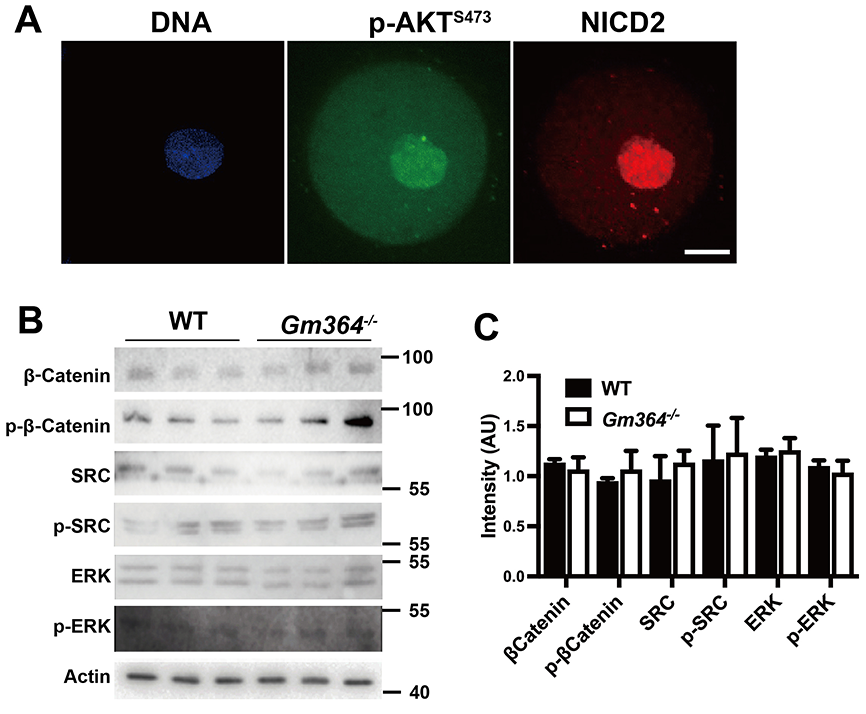

Supplement: Supplementary file 7 — Supplementary Figure 5 [file 41418_2021_861_MOESM7_ESM.tif]

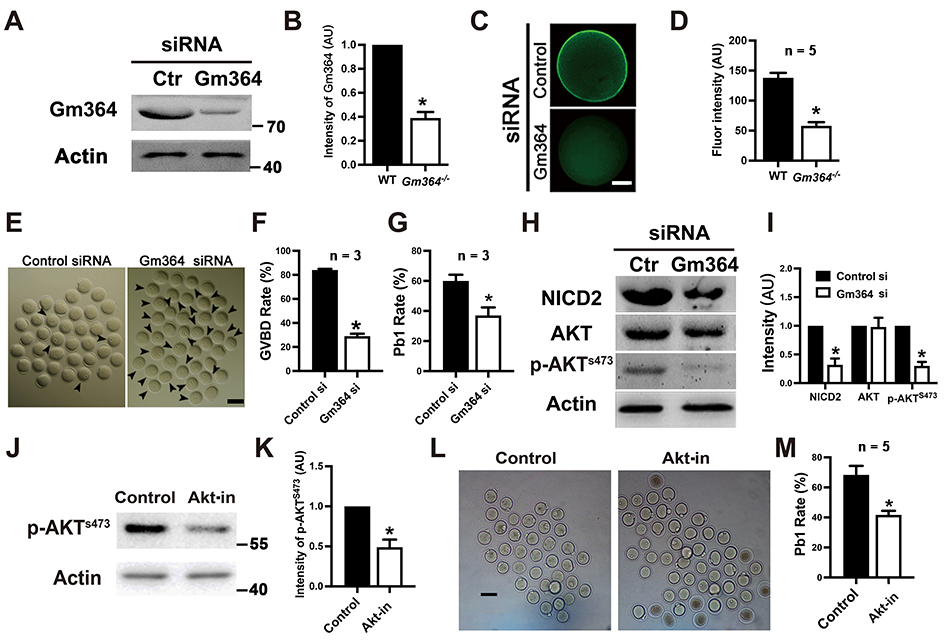

Supplement: Supplementary file 8 — Supplementary Figure 6 [file 41418_2021_861_MOESM8_ESM.tif]

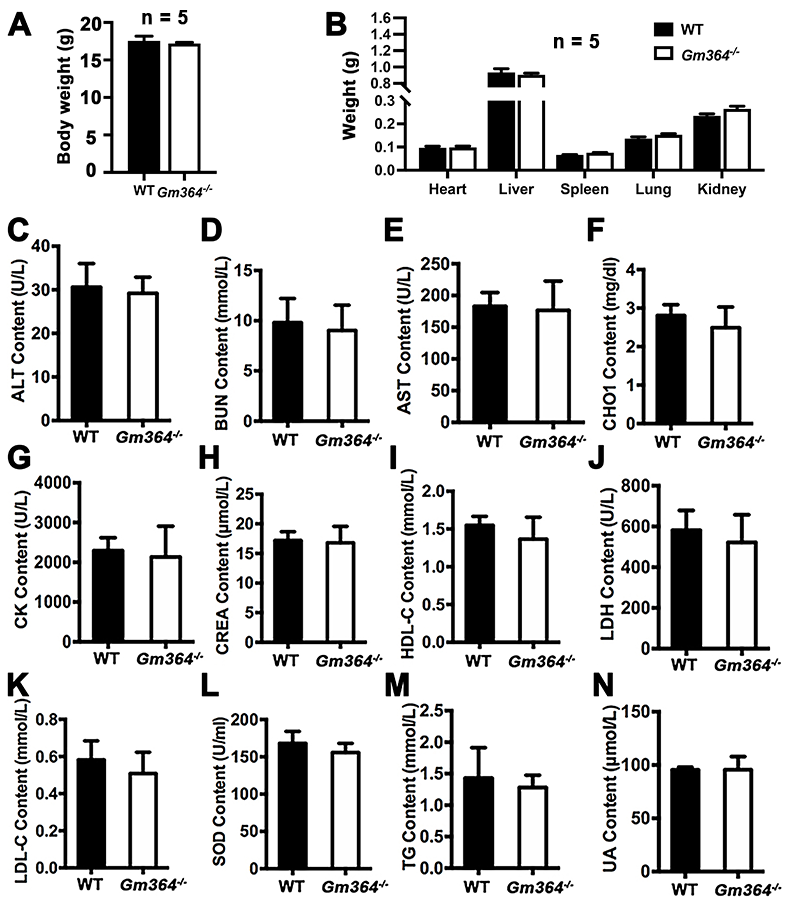

Supplement: Supplementary file 9 — Supplementary Figure 7 [file 41418_2021_861_MOESM9_ESM.tif]

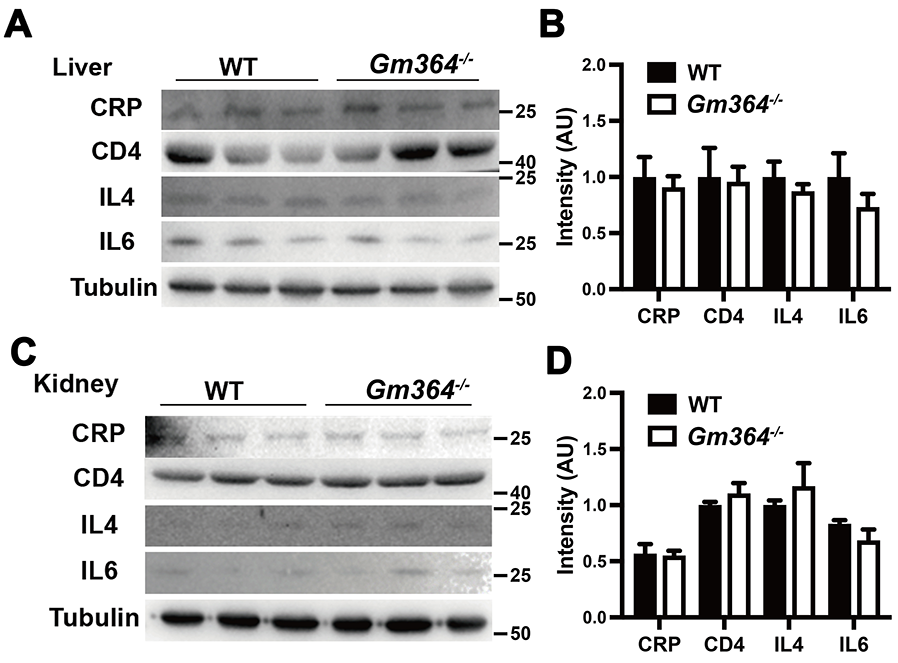

Supplement: Supplementary file 10 — Supplementary Figure 8 [file 41418_2021_861_MOESM10_ESM.tif]

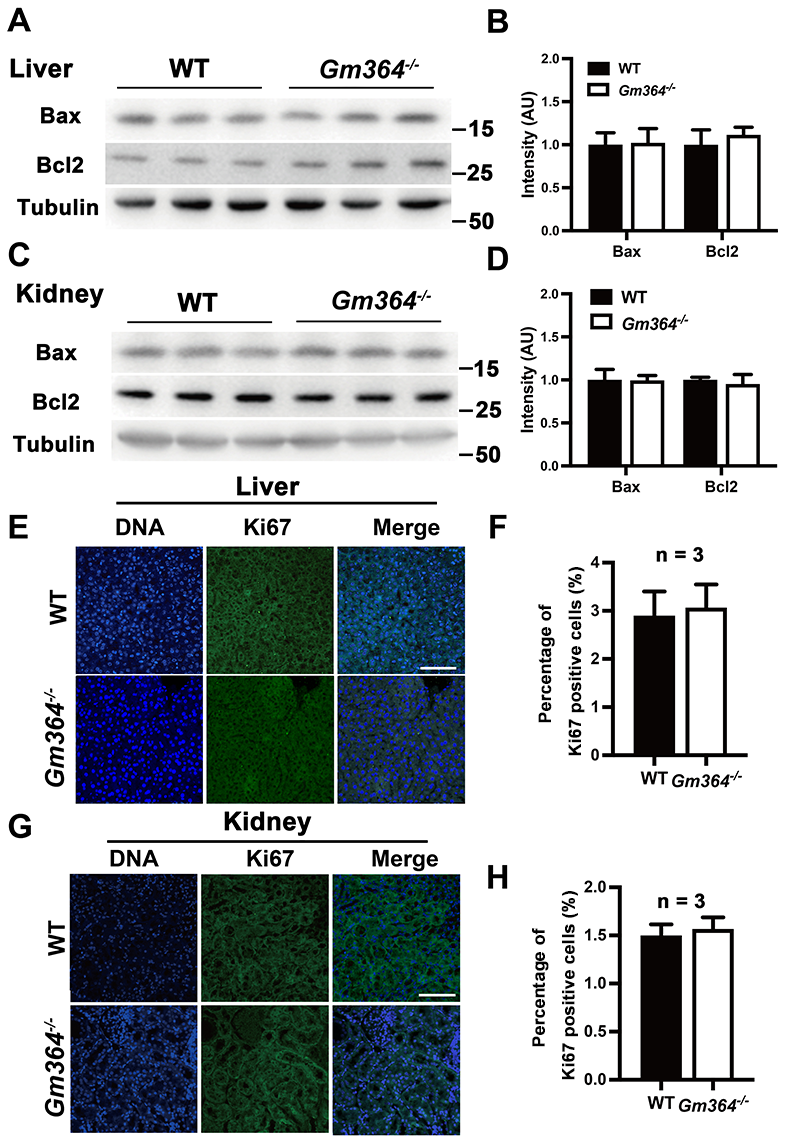

Supplement: Supplementary file 11 — Supplementary Figure 9 [file 41418_2021_861_MOESM11_ESM.tif]

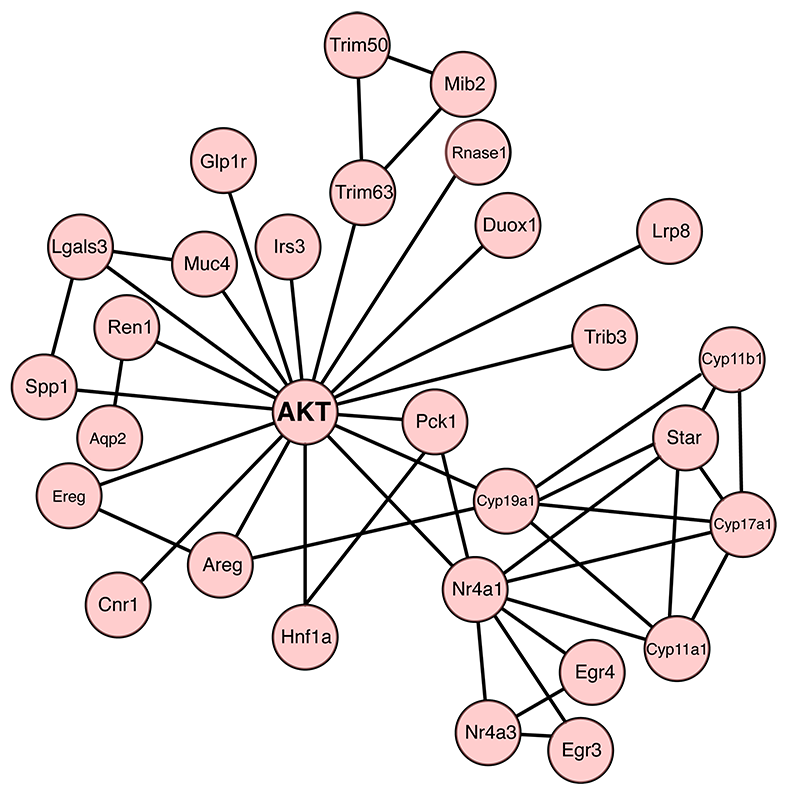

Supplement: Supplementary file 12 — Supplementary Figure 10 [file 41418_2021_861_MOESM12_ESM.tif]
